# Hypercrosslinked Polymers for Volatile and Very Volatile Organic Compound Capture Beyond Commercial Benchmarks

**DOI:** 10.1002/anie.202513362

**Published:** 2025-10-12

**Authors:** Paul Schweng, Elias Rippatha, Clemens Schwarzinger, Robert T. Woodward

**Affiliations:** ^1^ Institute of Materials Chemistry and Research, Faculty of Chemistry University of Vienna Währinger Straße 42 Vienna 1090 Austria; ^2^ Vienna Doctoral School in Chemistry University of Vienna Währinger Straße 42 Vienna 1090 Austria; ^3^ Institute of Chemical Technology of Organic Materials Johannes Kepler University Linz Altenbergerstraße 69 Linz 4040 Austria

**Keywords:** Adsorption, Hypercrosslinked polymer, Porous organic polymers, Separation and storage, Volatile organic compound

## Abstract

The effective capture of volatile organic compounds (VOCs) and very volatile organic compounds (VVOCs) is crucial for controlling indoor air quality, environmental monitoring, and emission regulation. However, effective VVOC adsorption remains challenging due to their low boiling points, high vapour pressures, and the susceptibility of many adsorbents to competitive water sorption. Here, we report a systematic study on the role of chemical functionality in hypercrosslinked polymers for (V)VOC capture under realistic operational conditions. A series of fluorene‐based hypercrosslinked polymers bearing heteroatom substituents (C, N, O, S, and SO_2_) was synthesised and characterised, exhibiting high thermal stability, surface areas up to 1600 m^2^·g^−1^, and micro‐/mesoporous architectures. The adsorption performance of these networks is evaluated using thermodesorption‐ and headspace‐gas chromatography–mass spectrometry. Under both dry and humid atmospheres, the hypercrosslinked polymers outperform a commercial benchmark sorbent in the uptake of a ten‐component (V)VOC mixture, with the amine‐functionalised network achieving the broadest analyte retention range even in the presence of water vapour. Our findings elucidate how polymer chemistry governs sorption behaviour and establish hypercrosslinked polymers as high‐performance, tuneable alternatives to state‐of‐the‐art commercial sorbents for capturing volatile analytes.

## Graphical abstract

The adsorption of volatile and very volatile organic compounds ((V)VOCs) is investigated using hypercrosslinked polymers with varied chemical functionalities. Mixed‐analyte breakthrough experiments reveal how polymer chemistry governs sorption under dry and humid conditions. Amine‐functionalised networks outperform commercial benchmarks, offering broad analyte retention, regenerability, and lower cost, establishing hypercrosslinked polymers as tuneable sorbents for (V)VOC capture.

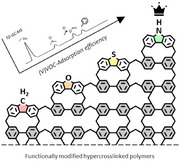

## Introduction

Volatile organic compounds (VOCs) from anthropogenic sources pose significant health and environmental risks, prompting the development of standardised methods for their quantification.^[^ ^1^, ^2^ ^]^ These protocols involve the trapping of potentially dangerous substance classes on sorbents, followed by thermal desorption and quantification via analytical detection.^[^ ^3^ ^]^ A particularly challenging subset of VOCs is very volatile organic compounds (VVOCs), defined by the possession of boiling points below 100 °C. The high volatility of VVOCs limits their effective adsorption on standard sorbents, drastically lowering breakthrough volumes. Furthermore, the adsorption efficiency of VVOCs is highly sensitive to sorbent polarity, necessitating the selection of appropriate sorbents, such as porous polymers, graphitised carbon blacks, or carbon molecular sieves. Commonly used porous polymer sorbents comprise nonpolar functional groups, limiting their analyte range, while more polar alternatives, such as graphitised carbon blacks, suffer from competitive water sorption, compromising performance.^[^ ^4^, ^5^ ^]^

Hypercrosslinked polymers (HCPs) are a versatile class of porous organic polymers characterised by their micro/mesoporous networks formed through extensive covalent crosslinking. Typically synthesised via Friedel–Crafts alkylation of aromatic precursors, HCPs can be constructed from a wide range of monomers and linking strategies, enabling the fine‐tuning of chemical functionality, pore structure, and surface polarity.^[^ ^6^ ^]^ The Friedel–Crafts chemistry used for HCP synthesis is well established in industrial practice and amenable to scale‐up.^[^ ^7^ ^]^ Examples of HCP scale‐up in the literature using batch reactors^[^ ^8^ ^]^ or continuous‐flow methods^[^ ^9^, ^10^ ^]^ further underscore the feasibility of translating these materials beyond laboratory scale. The ability for network fine‐tuning and their potential for scale‐up, coupled with exceptional thermal and chemical stabilities and high specific surface areas, make HCPs attractive candidates for diverse applications in adsorption and separation, such as (V)VOC capture.^[^ ^11^ ^]^

The tuneable chemistry and excellent textural properties of HCPs have enabled their deployment in various adsorption‐based separations. Amine‐functionalised HCPs have demonstrated enhanced CO_2_ capture capacities and selectivities via acid–base interactions with CO_2_ molecules.^[^ ^12^ ^]^ Likewise, the incorporation of polar or charged functional groups, such as protonated amines or sulfonates, has afforded selective uptake of organic dye pollutants from aqueous streams via electrostatic interactions, with reported capacities exceeding 3000 mg g^−1^ for anionic dyes.^[^ ^13^ ^]^ Sulfonated HCPs were also shown to be effective for atmospheric water harvesting, reaching total water uptake capacities of 81 wt % owing to their combination of network hydrophilicity and porosity.^[^ ^14^ ^]^ In heavy metal remediation, HCPs bearing ether or carbonate functionalities engage in metal–ligand coordination and cation–π interactions, facilitating efficient removal of Pb^2^⁺ and Cd^2^⁺ ions from wastewater.^[^ ^15^ ^]^ Sulfonated HCPs were also shown to be effective for the adsorption of St, Cs, and Ba ions in both batch adsorption processes^[^ ^16^ ^]^ and in flow‐through setups where the polymers were embedded in cellulose matrices to form hybrid membranes.^[^ ^17^ ^]^ Aniline‐based HCPs have also achieved benchmark iodine uptake from both vapour and aqueous phases, driven by charge‐transfer interactions with electron‐rich amine groups.^[^ ^18^ ^]^ These studies underscore the potential of HCPs as modular sorbents whose adsorption performance can be precisely tuned by synthetic design.

Despite the broad use of HCPs in separation and storage, their application to (V)VOC capture remains comparatively underexplored. Paul et al. demonstrated that tetraphenylmethyl‐based HCPs exhibit exceptional adsorption capacities for toluene (>150 wt %),^[^ ^19^ ^]^ outperforming benchmark sorbents such as zeolites, with CH/π interactions identified as key to their high affinity. Wang et al. reported a hydrophobic, non‐functional HCP that exhibited an exceptional benzene adsorption capacity of >17 mmol g^−1^ in both dry and humid conditions, outperforming activated carbon, zeolites, and the commercial resin XAD‐4.^[^ ^20^ ^]^ However, the efficient capture of VVOCs, characterised by high vapour pressures and low boiling points, remains a significant challenge due to their poor affinity for conventional sorbents and competitive moisture sorption. While hydrophobic HCPs perform well in the adsorption of apolar analytes such as toluene and benzene, they often fail to capture polar analytes; conversely, polar functional groups may enhance sorption of polar VVOCs but risk undesired water uptake. To date, there remains a limited understanding of how HCP chemistry governs the balance between analyte affinity and the competitive adsorption of undesired substrates. In particular, systematic studies that isolate the role of heteroatom‐based functional groups across otherwise identical HCP backbones are lacking.

Addressing these knowledge gaps requires analytical methods capable of monitoring subtle differences in sorbent performance under realistic operational conditions. Conventionally, adsorption performance is evaluated using single‐component isotherms. Although such measurements provide adsorption capacities and affinities, time‐intensive preparative steps limit broader analyte screening.^[^ ^21^ ^]^ Furthermore, practically relevant samples typically contain multiple components, resulting in competitive adsorption phenomena that are neglected in single‐component isotherms, leading to misleading selectivity values, a poor understanding of competitive adsorption effects, and the overestimation of adsorption capacities.^[^ ^22^ ^]^ In order to compare multiple sorbates simultaneously, chromatographic methods like gas chromatography–flame ionisation detection (GC‐FID) or thermodesorption–gas chromatography–mass spectrometry (TD‐GC‐MS) are used.^[^ ^23^, ^24^ ^]^ These analysis methods enable the simultaneous detection of multiple components with high sensitivity and short analysis times.

In this work, we present a systematic investigation of how the chemistry of HCPs influences the capture of multi‐component (V)VOC streams under realistic operating conditions. The implementation of new heteroatom functionalities and the lack of regioselectivity of Friedel–Craft alkylation can influence the 3D structure of HCPs.^[^ ^25^ ^]^ To minimise the influence of varying structural profiles between the polymers, we have maintained a constant monomer‐to‐linker ratio during synthesis to produce samples with similar porous properties, particle size distributions, and thermal degradation properties. Biphenyl‐based HCPs are synthesised using fluorene derivatives bearing distinct heteroatom functionalities (C, N, O, S, and SO_2_), allowing the influence of sorbent chemical composition on analyte‐sorbent interactions to be disentangled from variations in network topology. To probe adsorption efficacy, we employ a sensitive breakthrough analysis using TD‐GC‐MS, which enables the semi‐quantitative comparison of ten VOCs and VVOCs under both dry and humid conditions. Our integrated approach, combining tailored porous polymer design with multi‐component analysis, permits a nuanced understanding of how sorbent chemistry governs analyte affinity and breakthrough behaviour, particularly in the challenging VVOC regime, and reveals key advantages over conventional commercial materials.

## Results and Discussion

**Figure 1:**
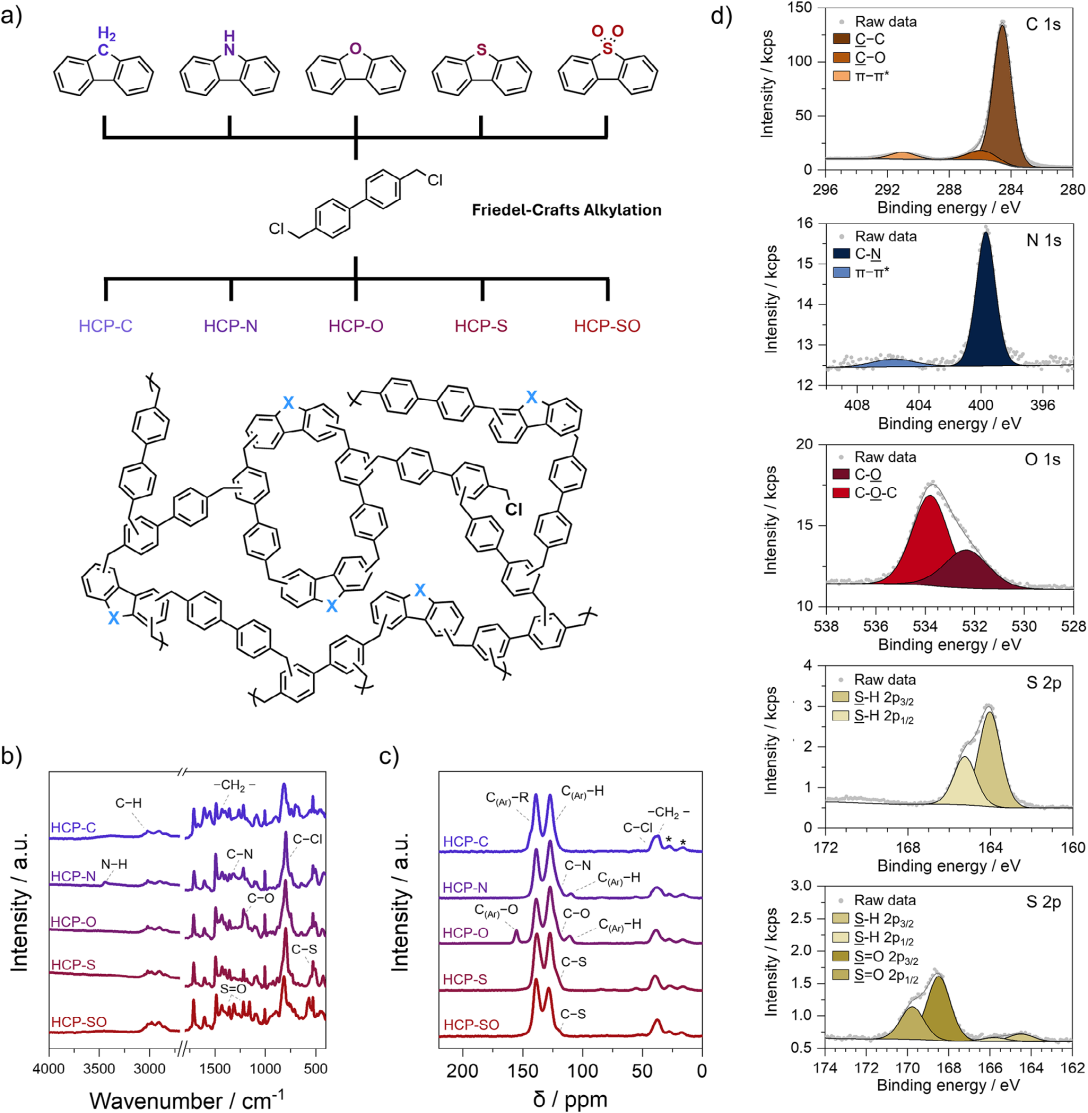
a) General reaction scheme for the synthesis of HCPs, b) FTIR spectra, c) ^13^C CP/MAS ssNMR spectra, d) representative high‐resolution XPS spectra (from top to bottom): C 1s of HCP‐C; N 1s of HCP‐N; O 1s of HCP‐O; S 2p of HCP‐S; and S 2p of HCP‐SO.

We synthesised five biphenyl‐based hypercrosslinked polymers containing fluorene derivatives comprising C, N, O, S, or SO_2_ substituents at the 9‐position via crosslinking with 4,4′‐bis(chloromethyl)‐1,1′‐biphenyl (Figure 1a). The polymers were washed with methanol and dried under vacuum at 80 °C. All networks were obtained in yields of > 70% (Table S1 and Figure S1) and are referred to as HCP‐X, where X denotes the chemical functionality. Detailed synthesis protocols are provided in the Supporting Information.

The hypercrosslinking of fluorene‐based compounds was confirmed with Fourier‐transform infrared spectroscopy (FTIR, Figure 1b), which showed characteristic bands for aromatic and aliphatic C─H stretching vibrations at 3020 and 2900 cm^−1^, respectively, in all samples. A peak at 1473 cm^−1^ was assigned to newly formed methylene crosslinks, and a band at 807 cm^−1^ was attributed to residual C─Cl bonds due to some incomplete conversion. For HCP‐N, signals at 3439 and 1315 cm^−1^ were attributed to N─H stretching vibrations of secondary amines and C─N stretching vibrations of aromatic amines, respectively. In HCP‐O, a prominent peak at 1198 cm^−1^ was assigned to C─O stretching vibrations of the aryl ether. Both HCP‐S and HCP‐SO exhibited C─S stretching vibrations at 567 cm^−1^. Additional signals at 1315 and 1157 cm^−1^, corresponding to asymmetric and symmetric S═O stretching vibrations of the sulfone group, were observed solely in HCP‐SO.

To further verify successful copolymerisation, we employed ^13^C cross‐polarisation/magic angle spinning solid‐state NMR (ssNMR, Figure 1c). In all HCPs, signals corresponding to newly formed methylene bridges are observed at around ∼38 ppm, while a weak shoulder peak at ∼42 ppm is attributed to residual C─Cl. Characteristic peaks for aromatic (C_Ar_–H) and substituted aromatic carbons (C_Ar_–R) are found throughout the set at ∼128 and ∼138 ppm, respectively. Line broadening occurs in ssNMR spectra due to anisotropic interactions and the absence of molecular tumbling, preventing the precise identification of heteroatom‐bound carbons in most cases. Fluorene analogues bearing C, S, or SO_2_ substituents were reported to show resonances at ∼135–140 ppm for carbons in the 4a,b positions, overlapping with the C_Ar_─R region.^[^ ^26^, ^27^ ^]^ In contrast, for N and O substituents, the 4a,b positions resonate at ∼120 ppm, overlapping with C_Ar_─H signals. However, we observed some shifting in the C_Ar_─H peaks in HCP‐N and HCP‐O, resulting in the appearance of additional signals at 110 and 155 ppm, respectively.

We employed X‐ray photoelectron spectroscopy (XPS) to probe the chemical composition of HCP surfaces (Figure 1d, Figures S4–S8 & Table S2). Throughout the set, high‐resolution C 1s spectra exhibited a peak at a binding energy of 284.8 eV, attributed to both aromatic and aliphatic C─C bonds. The inclusion of heteroatoms resulted in additional lower‐intensity peaks at 285.5 eV for C─N, 286.5 eV for C─O, and 285.6 eV for C─S. A broad π–π* shake‐up feature was observed at ∼291.0 eV in all spectra. High‐resolution O 1s spectra revealed a peak at 532.6 eV in all samples, attributed to C─O formed via the attack of the intermediate carbocation generated during polymerisation by trace water. The high‐resolution Cl 2p spectra confirmed the presence of residual C─Cl. The incorporation of carbazole into HCP‐N was confirmed by the high‐resolution N 1s spectrum, which showed a C─N peak at 399.7 eV. A broad satellite peak at 405.7 eV is attributed to a π–π* shake‐up feature, consistent with aromatic amines, as reported for polycarbazole and other conjugated nitrogenous polymers.^[^ ^28^, ^29^ ^]^ The presence of oxygen in HCP‐O was confirmed by the appearance of a peak at 533.8 eV in the high‐resolution O 1s spectrum, corresponding to C─O─C bonds, as well as the peak ascribed to C─O at 532.5 eV. The high‐resolution S 2p spectrum of HCP‐S revealed peaks at 164.0 eV and 165.2 eV, corresponding to 2p_3/2_ and 2p_1/2_, respectively, confirming the presence of C─S bonds. Signals appear shifted in HCP‐SO, displaying a typical asymmetric peak of a sulfone moiety with S 2p_3/2_ and 2p_1/2_ observed at 168.5 and 169.7 eV, respectively.

We used CHNS‐O elemental analysis (EA) to characterise the bulk chemical composition of the HCPs (Table S3), again confirming the incorporation of the fluorene analogues via increases in the corresponding heteroatom contents. The presence of O in all samples was confirmed by EA. Discrepancies between elemental compositions obtained via XPS and EA stem from several factors, including their respective detections of surface and bulk chemistries, atmospheric water adsorption prior to EA, and the inability of XPS to detect H.

**Figure 2:**
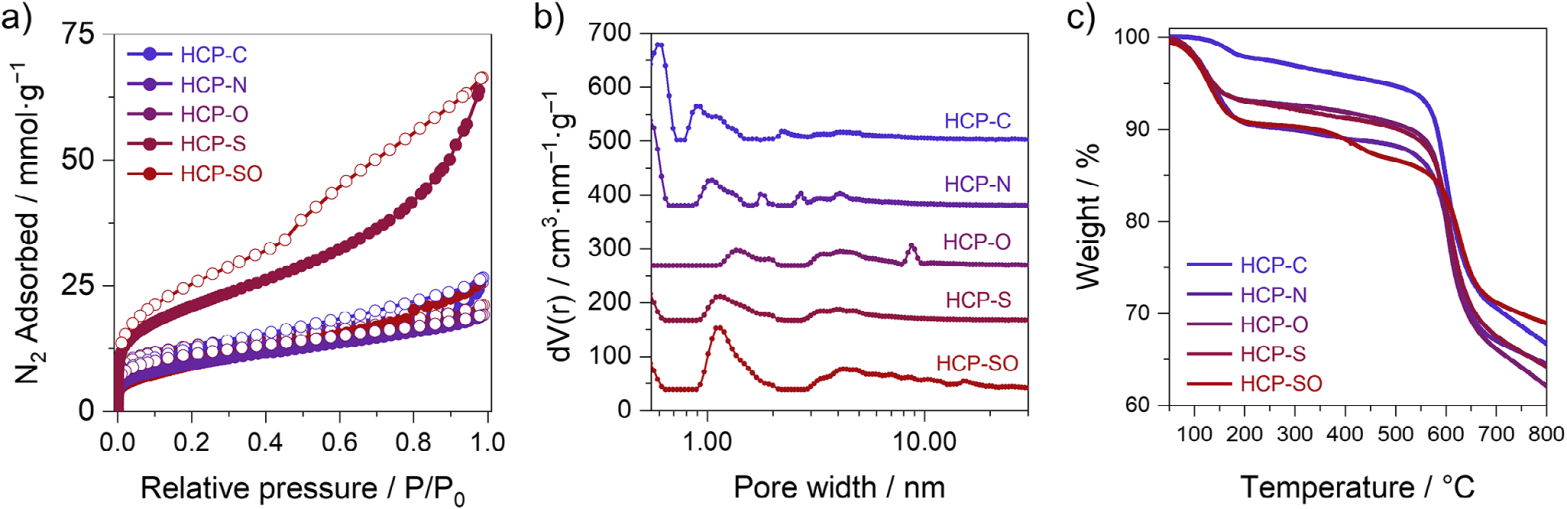
a) N_2_ adsorption–desorption isotherms of all HCPs measured at − 196 °C, b) QSDFT pore size distribution of all HCPs, and c) thermogravimetric curves of all HCPs recorded up to 800 °C at a ramp rate of 10 °C·min^−1^ under N_2_ flow.

We measured the N_2_ sorption isotherms of all HCPs at –196 °C to probe their textural properties (Figure 2a). All networks displayed elements of both type I and type IVa isotherms, indicative of micro‐ (<2 nm in diameter) and mesopores (2–50 nm in diameter). HCPs containing C, N, O, or S at the 9‐position of the fluorene monomer exhibited type H4 hysteresis, associated with narrow slit‐like mesopores, while HCP‐SO showed H2 hysteresis, indicative of a broader pore size distribution and restricted pore necks. Quenched solid density functional theory (QSDFT) pore size distributions confirmed the heterogeneity of pore structures throughout HCPs, with pore volume deriving from micropores and mesopores of up to 10 nm in diameter (Figure 2b). The pore volume of each sample was dependent on the degree of hypercrosslinking within the polymer network.^[^ ^30^ ^]^ As HCP‐C and HCP‐N display almost equivalent pore size distributions, differences in (V)VOC adsorption can be attributed to the influence of co‐monomer composition in the polymer network. All networks exhibited BET‐specific surface areas (SSA_BET_) exceeding 700 m^2^·g^−1^, with HCP‐SO displaying the highest SSA_BET_ of 1629 m^2^·g^−1^. The SSA_BET_, micropore volume, and total pore volume of all HCPs are provided in Table 1.

**Table 1:** Summary of textural properties of HCP‐C, HCP‐N, HCP‐O, HCP‐S, and HCP‐SO. Data includes BET specific surface area, SSA_BET_, volume of micropores, V_MICRO_, and total pore volume, V_TOT_.

HCP network	SSA_BET_ (m^2^·g^−1^)	V_MICRO_ (cm^3^·g^−1^)	V_TOT_ (cm^3^·g^−1^)
HCP‐C	982	0.25	0.77
HCP‐N	812	0.19	0.66
HCP‐O	721	0.10	0.83
HCP‐S	740	0.17	0.61
HCP‐SO	1629	0.31	2.07

Thermal desorption (TD), when coupled to GC‐MS, enables the sensitive analysis of analytes released from sorbent substrates upon heating. To probe analyte retention effectively, the sorbent must withstand temperatures of up to 300 °C during TD.^[^ ^3^ ^]^ The degradation temperature of each sorbent was determined by TGA (Figure 2c and Figure S9 for the commercial sorbent). In all cases, weight loss below 200 °C was attributed to the removal of adsorbed water. Network HCP‐SO exhibited a reduced thermal stability with a degradation onset of about 350 °C and was further evaluated by TGA‐FTIR and compared to HCP‐S (Figure S10). At 350 °C, we observed the emission of water from HCP‐SO, which was not present in HCP‐S. Further thermal degradation of the polymers was not observed by TGA‐FTIR, however signals for released SO_2_ may be masked by those for water. Although HCP‐SO's excellent porosity and outstanding polar properties show its potential as an efficient sorbent for the capture of water or (V)VOCs under dry conditions, the remaining polymers herein, including the commercial sorbent, were thermally stable up to an onset of about 540 °C. Thermally mild desorption and extraction techniques, like those used in vacuum‐assisted sorbent desorption (VASE)^[^ ^31^ ^]^ or solvent extraction,^[^ ^32^ ^]^ are available and could enable the utilisation of HCP‐SO. However, as HCP‐SO appeared not to withstand the required temperatures for analyte desorption/activation, it was excluded from further assessment. Before the adsorption experiments, all sorbents were activated under nitrogen flow at 300 °C for 6 h. To verify the removal of residual contaminants or degradation products, the activated HCPs were immediately analysed by TD‐GC‐MS and displayed negligible levels of contamination (Figure S11) and no changes upon visual inspection (Figure S12). A sorbent column was considered fully activated if less than 10% of all integrated peak areas were assigned to interferents.

A set of ten (V)VOCs characterised by a broad range of individual physicochemical properties (molar volume, boiling point, water–octanol partition coefficient (LogP), Hansen solubility parameters, and vapour pressure) was chosen, as these parameters are known to influence sorption behaviour (Table S4). The setup for the adsorption experiments consisted of two sorbent columns and a cooled headspace‐vial connected in series, where each column contained the same sorbent (Figures S2 and S3). A sum standard of ten (V)VOCs was injected once for each sorbent into the first sorbent column in either a nitrogen or an air carrier stream. Analytes that interacted strongly with the stationary phase are retained in the first column, while compounds with a higher breakthrough potential are either trapped in the second column or are condensed in the headspace vial. After adsorption, both sorbent columns were immediately analysed with TD‐GC‐MS (Figure S13) and the condensed phase with headspace‐GC‐MS (HS‐GC‐MS) (Figure S14). A detailed protocol can be found in the Supporting Information. Repeatability of the analysis method was established by injecting the analytes into the column with the commercial sorbent three times (Figure S15).

**Figure 3:**
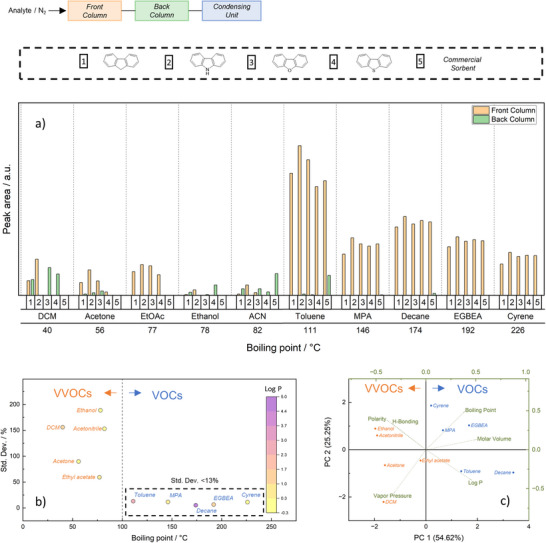
a) TD‐GC‐MS results of breakthrough measurements, characterising four HCPs (1–4, monomer units shown) and the commercial sorbent (5). Analytes (ethanol, acetonitrile (ACN), dichloromethane (DCM), ethyl acetate, toluene, 1‐methoxypropan‐2‐yl acetate (MPA), decane, 2‐butoxyethyl acetate (EGBEA), and dihydrolevoglucosenone (cyrene)) were described according to their detector areas in two sorbent columns connected in series (front and back column). b) Standard deviation determined from the peak area found in the front column of each analyte. The analytes were divided into two groups and characterised based on their respective standard deviation (VVOCs and VOCs) and labelled with their respective Log P (octanol/water coefficient). High coefficients can be assigned to less polar and more lipophilic substances like decane, while lower coefficient numbers, like that of ethanol, tend to be more polar and hydrophilic. The analytes were divided into two groups and characterised based on their respective standard deviation (VVOCs and VOCs). c) Principal component analysis of six physico‐chemical parameters of VVOCs and VOCs.

Figure 3a displays the integrated peak areas of individual analytes in the first and second columns for all sorbents, analysed by TD‐GC‐MS. By calculating the standard deviation of individual analytes across all peak areas (i.e., all adsorbent materials) found in the first column, the influence of physicochemical parameters on sorbate/sorbent interaction is unveiled (Figure 3b). Analytes belonging to VOCs (boiling point > 100 °C) showed nearly constant peak areas when analysing the front column, leading to low standard deviations (<13%) and, therefore, successful sorption on all stationary phases is concluded (Figure 3a,b). Despite having a high vapour pressure and volatility, toluene was fully captured on all HCPs, yet showed early breakthrough when using the commercial sorbent. Overall, all VOCs were successfully enriched on each sorbent, resulting in low standard deviations across all measurements, i.e., VOC adsorption appeared independent of the varying physicochemical properties of HCP sorbents.

Analytes with boiling points below 100 °C (VVOCs) showed comparably larger differences in peak areas when measuring the front column, thus increasing overall standard deviations (Figure 3a,b). To estimate sorption behaviour and to account for larger standard deviations of VVOCs not solely attributable to volatility, physicochemical properties such as polarity (octanol/water coefficient) are used. Although the octanol–water coefficient and boiling point for ethanol and acetonitrile are similar to those of acetone, the standard deviation of these compounds was higher. Further physicochemical properties of all analytes influencing adsorption (Hansen solubility parameters: polarity and H‐Bonding, boiling point, molar volume, vapor pressure, and log P) were statistically assessed with a principal component analysis (PCA) biplot (Figure 3c). PCA was used to reduce the number of variables (six physicochemical parameters) into two principal components (PC1 and PC2) based on a linear combination of the variables. The cumulative variance of PC1 (*x*‐axis) and PC2 (*y*‐axis) is almost 80%, indicating a high information retention of the dataset. Spatial clustering of (V)VOCs represents strong similarities, while vectors starting from the point of origin represent variables. Vectors with a longer distance to the point of origin contribute a stronger effect on PC1 and/or PC2. Depending on the direction of the vector, they can show positive (similar direction) or negative correlation (opposite direction) with one another. PCA reveals that ethanol and acetonitrile have strong potential for either forming hydrogen bonds or exhibiting high polarity, while sharing similar properties in all other variables. Strong adsorption and an increased analyte range can therefore only be achieved if a polar/hydrophilic chemical functionalisation is incorporated into the sorbent. Additionally, the structure of carbon‐rich polymers promotes hydrophobic effects, π–π and π–electron donor–acceptor interactions, leading to the efficient adsorption of nonpolar aromatic substances and thus making the synthesised HCPs strong sorbents for both polar and nonpolar analytes.

**Figure 4:**
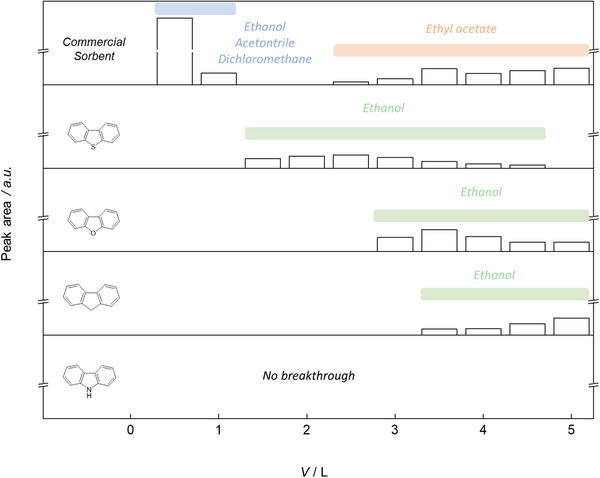
Headspace‐GC‐MS analysis of the post‐sorbent cold trap of four HCPs (monomer units shown) and the commercial sorbent.

The (V)VOCs with high breakthrough potential were condensed in a headspace‐vial cooled with an acetone / liquid nitrogen mixture, connected behind the two sorbent columns, and analysed with headspace‐GC‐MS (Figure 4). During fractionated analysis, the acetone/nitrogen bath used for cooling led to some contamination within the headspace vials. Acetone is therefore excluded from further discussion, as it was also detected in blank measurements. HS‐GC‐MS data reveal that most VVOCs did not adsorb to the commercial sorbent and were eluted at purge volumes between 0.5 and 1 L. As ethyl acetate has a lower volatility compared to the other analytes, it was detected at volumes between 3.5 and 5 L. The poor retention of VVOCs in the commercial sorbent was expected due to its nonpolar network with negligible functionality.

To compare the adsorption efficacy of the HCP sorbents, the breakthrough percentage was determined as the ratio of the detector intensity of the back column to the combined detector intensities of both the back and front columns (Table 2). The weakest analyte retention was observed in the commercial sorbent, whereas all HCPs, particularly HCP‐N, captured higher quantities of analyte. Ethanol was the only analyte that was detected in most cold trap measurements (Figure 4). While ethanol was already detected at the lowest volume fractions (0.5–1 L) with the commercial sorbent, breakthrough volumes > 1.5 L were measured for all HCPs. Sorbents HCP‐S and HCP‐O showed the first breakthrough of ethanol at volumes of 1.5–3 L, while HCP‐C had only partial breakthrough from the first column, resulting in delayed detection after 3.5 L. This effect is also visible in Figure 4, as purge volumes shift to higher values for HCP‐C. Nevertheless, all other analytes were retained by the second sorbent column, as no other compounds were found in the cold traps. The reuseability of the best‐performing sorbent (HCP‐N) was assessed by regeneration of the network after adsorption via activation at 300 °C, after which the network was then exposed to a fresh analyte mixture. Five cycles of adsorption and regeneration were performed, resulting in a low standard deviation (5.2%) across cycles, showcasing the excellent regenerability of HCP‐N (the retention of cyrene for each cycle is shown in Figure S16).

**Table 2:** Breakthrough of ten analytes (ethanol, acetonitrile (ACN), dichloromethane (DCM), ethyl acetate, toluene, 1‐methoxypropan‐2‐yl acetate (MPA), decane, 2‐butoxyethyl acetate (EGBEA) and dihydrolevoglucosenon (cyrene)) in a commercial sorbent and four HCPs (monomer units shown) under dry inert (0% relative humidity (RH), nitrogen (N_2_)) and humid conditions under technical air (50% RH, air). Columns are marked according to their breakthrough in green (no breakthrough: 0.00%), yellow (partial breakthrough: 5.00%–99.99%) and red (complete breakthrough: 100.00%). Hansen solubility parameters (HSP) for polarity (δ_P_) and H‐bonding (δ_H_) were calculated for each fluorene‐based monomer.

Analyte	Commercial sorbent		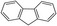 HSP: δ_P_: 2.8 MPa^0.5^ δ_H_: 2.8 MPa^0.5^	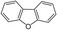 HSP: δ_P_: 4.0 MPa^0.5^ δ_H_: 3.2 MPa^0.5^	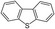 HSP: δ_P_: 3.3 MPa^0.5^ δ_H_: 4.5 MPa^0.5^	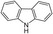 HSP: δ_P_: 6 MPa^0.5^ δ_H_: 5.4 MPa^0.5^	
	0% RH, N_2_	50% RH, Air	0% RH, N_2_	0% RH, N_2_	0% RH, N_2_	0% RH, N_2_	50% RH, Air
Ethanol	100.00	100.00	78.25	100.00	100.00	0.00	0.00
Acetone	100.00	100.00	8.92	23.47	0.00	9.14	0.00
Acetonitrile	100.00	100.00	84.72	71.20	100.00	0.00	0.00
DCM	100.00	100.00	51.80	100.00	100.00	0.00	0.00
Ethyl acetate	100.00	79.57	0.00	0.00	0.00	0.00	0.00
Toluene	14.78	11.45	0.00	0.45	0.00	0.81	0.00
MPA	0.84	0.00	0.00	0.00	0.00	0.00	0.00
Decane	2.52	1.03	0.00	0.00	0.00	0.00	0.00
EGBEA	0.00	0.00	0.00	0.00	0.00	0.00	0.00
Cyrene	0.00	0.00	0.00	0.00	0.00	0.00	0.00
No. of analytes with no breakthrough	4	4	6	6	7	9	10
No. of analytes with breakthrough	6	6	4	4	3	1	0

The lack of regioselectivity during crosslinking of HCPs makes computer modelling of polymer/analyte interactions challenging.^[^ ^33^ ^]^ The availability and simplicity of Hansen solubility parameters (HSP) render them a powerful tool for describing physical interactions between molecules. In inverse gas chromatography, retention volumes are used to predict HSP, showing the strong relation between HSP and physical interactions between stationary phases and volatiles.^[^ ^34^ ^]^ While the quantitative prediction of these interactions remains impossible, relative predictions can be made, especially for compounds with highly symmetric shapes and no potential for tautomerisation.^[^ ^35^ ^]^ As this applies to the co‐monomers used, we have investigated their HSP in the context of (V)VOC adsorption. To understand the interactions between sorbate and HCP sorbents, physicochemical parameters of the fluorene‐derived monomers used during synthesis were calculated employing the Yamamoto molecular break method (Tables 2 and S4).^[^ ^36^ ^]^ By comparing calculated data with experimental results, an increase in surface area, polarity and potential for hydrogen bonding of heteroatoms in HCPs is found to result in a drastic increase in the analyte sorption range. More polar chemical functional groups benefit the adsorption of very volatile compounds, but may lead to unwanted side effects such as water trapping.^[^ ^4^, ^5^ ^]^ Since the removal of VOCs from surfaces is highly dependent on gas flow, humidity and temperature, we compared the commercial sorbent with HCP‐N under 50% relative humidity (RH) with technical air at room temperature. These standardised conditions are typically used when evaluating emission rates of building products.^[^ ^23^ ^]^ Both polymers showed similar adsorption results as under dry conditions (Table 2), which was anticipated as the commercial sorbent does not have any hydrophilic functional groups and water adsorption for HCP‐N was insignificant < 50% RH (Figure S17). Comparison of HCP performance against a broader set of established commercial sorbents such as specialist polymers, activated carbons, and zeolites for (V)VOC capture revealed that HCP‐N exhibited the best overall performance for the selected substrate mixture, even in the presence of water vapour (Table S5).^[^ ^23^, ^32^, ^37^, ^38^ ^]^ The pores between 1–2 nm in diameter of the HCPs benefit adsorption capacities as the kinetic diameter of strong volatiles like ethanol, acetone and acetonitrile is ≤ 0.5 nm, while diameters of bigger compounds such as toluene can be almost twice as high.**^[^** ^39^, ^40^ **^]^** Overall, by implementing a free secondary amine, the analyte range was increased under competitive adsorption, while hitting the sweet‐spot for maintaining minimal water retention.

Finally, we conducted a cost comparison of our HCPs and a selection of leading commercial materials for (V)VOC capture (Table S6, calculation detailed in the Supporting Information). Our leading material, HCP‐N, was at least four times cheaper than all of the commercial sorbents. We would, however, like to emphasise that the calculated cost for these materials is for comparative purposes only and considers lab‐scale production.

## Conclusion

We have demonstrated that the chemical functionality of hypercrosslinked polymers (HCPs) plays a key role in the selective capture of volatile and very volatile organic compounds ((V)VOCs) under realistic operating conditions. We systematically varied organic substituents (C, N, O, S, and SO_2_) in fluorene‐derived HCPs and evaluated their performance via breakthrough analysis using a multi‐component TD‐GC‐MS and headspace‐GC‐MS. While all polymers exhibited excellent porous properties, we show that polar functionalities significantly enhance the adsorption of challenging (V)VOCs. Notably, the amine‐decorated HCP (HCP‐N) achieved the broadest analyte retention profile, outperforming a commercial benchmark adsorbent even under humid conditions (50% RH), balancing enhanced sorption of polar analytes with limited water uptake. Furthermore, the regeneration and reuse of HCP‐N over five cycles was demonstrated, with no detriment to adsorbent performance. These findings establish functionalised HCPs not merely as promising alternatives to commercial adsorbents, but as demonstrably superior materials for (V)VOC capture across a range of operational environments, offering high‐performance solutions for volatile analyte sorption under real‐world conditions. By extending the analysis beyond traditional single‐component isotherms to encompass complex, multi‐component (V)VOC mixtures, this study establishes a framework for assessing sorbent performance under conditions that more closely resemble practical emission scenarios.

## Supporting Information

The experimental section and further details, as well as tables and graphs, are presented in the Supporting Information.

## Conflict of Interests

The authors declare no conflict of interest.

## Supporting information



Supporting Information

## Data Availability

The data that support the findings of this study are available from the corresponding author upon reasonable request.

## References

[1] C. Duan , H. Liao , K. Wang , Y. Ren , Environ. Res. 2023, 216, 114386. 36162470 10.1016/j.envres.2022.114386

[2] ISO16000‐1 2004.

[3] ISO16000‐6 2011.

[4] T. Salthammer , Indoor Air 2016, 26, 25–38. 25471461 10.1111/ina.12173

[5] M. Even , E. Juritsch , M. Richter , Trends Anal. Chem. 2021, 140, 116265.

[6] L. Tan , B. Tan , Chem. Soc. Rev. 2017, 46, 3322–3356. 28224148 10.1039/c6cs00851h

[7] D. J. Macquarrie , in Catalytic Asymmetric Friedel‐Crafts Alkylations (Eds: M. Bandini , A. Umani‐Ronchi , G. A. Olah ), Wiley‐VCH‐Verl., Weinheim 2009, pp. 271–288.

[8] V. Rozyyev , D. Thirion , R. Ullah , J. Lee , M. Jung , H. Oh , M. Atilhan , C. T. Yavuz , Nat. Energy 2019, 4, 604–611.

[9] N. Chanchaona , L. Ding , S. Lin , S. Sarwar , S. Dimartino , A. J. Fletcher , D. M. Dawson , K. Konstas , M. R. Hill , C. H. Lau , J. Mater. Chem. A 2023, 11, 9859–9867.

[10] N. Chanchaona , C. H. Lau , Ind. Eng. Chem. Res. 2023, 62, 9046–9053.

[11] Y. Ahmadi , K.‐H. Kim , Polym. Rev. 2023, 63, 365–393.

[12] M. R. Moradi , A. Torkashvand , H. Ramezanipour Penchah , A. Ghaemi , Sci. Rep. 2023, 13, 9214. 37280347 10.1038/s41598-023-36434-4 PMC10244359

[13] P. Su , X. Zhang , Z. Xu , G. Zhang , C. Shen , Q. Meng , New J. Chem. 2019, 43, 17267–17274.

[14] P. Schweng , F. Mayer , D. Galehdari , K. Weiland , R. T. Woodward , Small 2023, 19, e2304562. 37621031 10.1002/smll.202304562

[15] H. Masoumi , A. Ghaemi , Sci. Rep. 2024, 14, 4817. 38413656 10.1038/s41598-024-54430-0 PMC11315691

[16] A. M. James , S. Harding , T. Robshaw , N. Bramall , M. D. Ogden , R. Dawson , ACS Appl. Mater. Interfaces 2019, 11, 22464–22473. 31141662 10.1021/acsami.9b06295 PMC7007012

[17] F. Mayer , P. Schweng , S. Braeuer , S. Hummer , G. Koellensperger , A. Mautner , R. Woodward , A. Bismarck , Small Sci 2024, 4, 2400182. 40212259 10.1002/smsc.202400182 PMC11935042

[18] B. Liu , C. Mao , Z. Zhou , Q. Wang , X. Zhou , Z. Liao , R. Deng , D. Liu , J. Beiyuan , D. Lv , J. Li , L. Huang , X. Chen , W. Yuan , Int. J. Mol. Sci. 2022, 24, 370. 36613814 10.3390/ijms24010370 PMC9820307

[19] G. Paul , F. Begni , A. Melicchio , G. Golemme , C. Bisio , D. Marchi , M. Cossi , L. Marchese , G. Gatti , ACS Appl. Polym. Mater. 2020, 2, 647–658.

[20] J. Wang , W.‐Q. Wang , Z. Hao , G. Wang , Y. Li , J.‐G. Chen , M. Li , J. Cheng , Z.‐T. Liu , RSC Adv. 2016, 6, 97048.

[21] T. Ashirov , J. Lim , A. Robles , T. Puangsamlee , P. W. Fritz , A. Crochet , X. Wang , C. Hewson , P. Iacomi , O. Š. Miljanić , A. Coskun , Angew. Chem. Int. Ed. 2025, 64, e202423809. 10.1002/anie.202423809 39804699

[22] H. Rajabi , M. Hadi Mosleh , T. Prakoso , N. Ghaemi , P. Mandal , A. Lea‐Langton , M. Sedighi , Chemosphere 2021, 283, 131288. 34182650 10.1016/j.chemosphere.2021.131288

[23] M. Richter , E. Juritsch , O. Jann , J. Chromatogr. A 2020, 1626, 461389. 32797860 10.1016/j.chroma.2020.461389

[24] X. Huang , M. Tang , H. Li , L. Wang , S. Lu , Chemosphere 2023, 313, 137513. 36495972 10.1016/j.chemosphere.2022.137513

[25] F. Begni , F. Gullo , G. Paul , R. Rea , M.‐C. Ferrari , E. Mangano , M. Cossi , G. Gatti , L. Marchese , ACS Appl. Polym. Mater. 2022, 4, 5281–5286.

[26] J. Hu , S. Yang , X. Wang , D. Zhang , B. Tan , Macromolecules 2024, 57, 5507–5519.

[27] R. V. Law , D. C. Sherrington , C. E. Snape , I. Ando , H. Kurosu , Macromolecules 1996, 29, 6284–6293.

[28] H. F. Lee , J. A. Gardella , Polymer 1992, 33, 4250–4259.

[29] S. Hino , M. Nakazato , K. Matsumoto , Chem. Phys. 1988, 127, 411–417.

[30] Z. Liu , T. Yang , Y. Song , N. Zhao , S. Wu , Z. Ma , X. Gong , X. Tian , Z. Liu , RSC Appl. Polym. 2025, 3, 746.

[31] P. T. Justen , M. L. Kilpatrick , J. L. Soto , S. D. Richardson , Environ. Sci. Technol. 2024, 58, 1321–1328. 38159052 10.1021/acs.est.3c07097

[32] Y. Miyake , M. Tokumura , Q. Wang , Z. Wang , T. Amagai , Air Qual Atmos Health 2017, 10, 737–746. 28936271 10.1007/s11869-017-0465-0 PMC5581818

[33] A. Madanchi , E. Azek , K. Zongo , L. K. Béland , N. Mousseau , L. Simine , ACS Physical Chemistry Au 2025, 5, 3–16. 39867446 10.1021/acsphyschemau.4c00063 PMC11758375

[34] K. Adamska , R. Bellinghausen , A. Voelkel , J. Chromatogr. A 2008, 1195, 146–149. 18502435 10.1016/j.chroma.2008.05.020

[35] A. Benazzouz , L. Moity , C. Pierlot , V. Molinier , J.‐M. Aubry , Colloids Surf. A 2014, 458, 101–109.

[36] S. Abbott , H. Yamamoto , Hansen Solubility Parameters in Practice, Hansen‐Solubility.com, Denmark 2015.

[37] Y.‐T. Zhao , L.‐Q. Yu , X. Xia , X.‐Y. Yang , W. Hu , Y.‐K. Lv , Anal. Methods 2018, 10, 4894–4901.

[38] A. Schieweck , J. Gunschera , D. Varol , T. Salthammer , Anal. Bioanal. Chem. 2018, 410, 3171–3183. 29594428 10.1007/s00216-018-1004-z PMC5910464

[39] C. D. Baertsch , H. H. Funke , J. L. Falconer , R. D. Noble , J. Phys. Chem. 1996, 100, 7676–7679.

[40] X. Li , Y. Liu , Q. Liu , Z. Zheng , H. Guo , RSC Adv. 2022, 12, 7189. 35424694 10.1039/d1ra09061e PMC8982167

