# Supplementary material for: Hypercrosslinked Polymers for Volatile and Very Volatile Organic Compound Capture Beyond Commercial Benchmarks
Source: Angew Chem Int Ed Engl. 2025 Oct 12;64(48):e202513362. doi: 10.1002/anie.202513362 (PMC12643350; doi:10.1002/anie.202513362)
Supplement: Supplementary file 1 — Supporting Information [file ANIE-64-e202513362-s001.docx]

**Supporting Information**

**Hypercrosslinked polymers for volatile and very volatile organic compound capture beyond commercial benchmarks**

Paul Schweng,‡^a,c^ Elia Rippatha,‡^b^ Clemens Schwarzinger,^b^* Robert T. Woodward^a^*

^a^ Institute of Materials Chemistry and Research, Faculty of Chemistry, University of Vienna, Währinger Straße 42, 1090, Vienna, Austria

^b^ Institute for Chemical Technology of Organic Materials, Johannes Kepler University Linz, Altenbergerstraße 69, 4040, Linz, Austria

^c^ Vienna Doctoral School in Chemistry, University of Vienna, Währinger Straße 42, 1090, Vienna, Austria

‡ These authors contributed equally

*Corresponding author:
E-mail: clemens.schwarzinger@jku.at
E-mail: robert.woodward@univie.ac.at

**1. Experimental methods**

**1.1. Materials**

All chemicals were obtained from commercial sources and used without further purification. Fluorene (98%), carbazole (synthesis grade), dibenzofuran (synthesis grade), dibenzothiophene (98%), dibenzothiophenone sulfone (97%), 4,4′-bis(chloromethyl)-1,1′-biphenyl (95%), FeCl₃ (reagent grade, 97%), and 1,2-dichloroethane (ACS reagent, ≥99%) were purchased from Sigma-Aldrich. Methanol (≥99.8%) was purchased from Fisher Scientific. All solvents used for VOC measurements were acquired from common chemical retailers with purities ≥97%. Nitrogen (4.6) and technical air (hydrocarbon-free) were sourced from Linde. The commercial sorbent is a porous polymer system with a mesh size of 60/80 and was provided by the producer. For confidentiality reasons, further details regarding the composition and origin of the commercial sorbent are not disclosed.

**1.2. Synthesis of hypercrosslinked polymers**

Hypercrosslinked polymers were synthesised by dissolving the crosslinker, 4,4′-bis(chloromethyl)-1,1′-biphenyl (2.512 g, 10 mmol) and the monomer, either fluorene (0.831 g, 5 mmol), carbazole (0.836 g, 5 mmol), dibenzofuran (0.841 g, 5 mmol), dibenzothiophene (0.921 g, 5 mmol), or dibenzothiophenone sulfone (1.081 g, 5 mmol) in 1,2-dichloroethane (25 mL) and stirred at room temperature. Upon dissolution, FeCl_3_ (1.622 g, 10 mmol) was added over stirring, and the reaction mixture was heated at 80 °C overnight. The resulting solids were washed multiple times with methanol, followed by an additional 24 h wash with methanol in a Soxhlet apparatus. Excess methanol was removed by allowing the polymers to air-dry at room temperature in a fume hood for several hours, followed by drying in an oven at 80 °C overnight. The networks were gently ground with a pestle and mortar, yielding the final products as a fine powder (images shown in Figure S1 and yields are provided in Table S1).


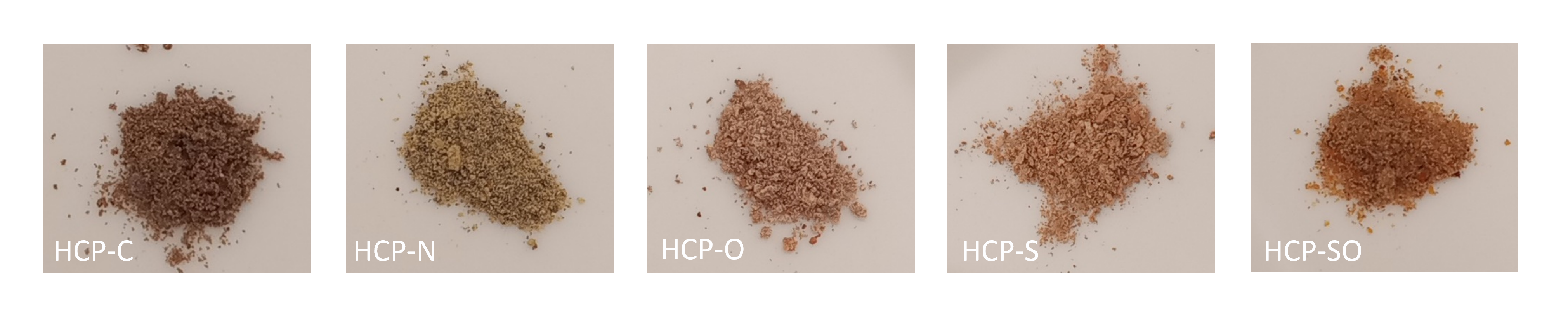
**Figure S1.** Photographs of all HCPs

**Table S1.** Yield of all HCPs

|  | Monomer | Yield (g) | Yield (%) |
| --- | --- | --- | --- |
| HCP-C | Fluorene | 2.80 | 107 |
| HCP-N | Carbazole | 2.37 | 91 |
| HCP-O | Dibenzofuran | 2.49 | 95 |
| HCP-S | Dibenzothiophene | 2.70 | 100 |
| HCP-SO | Dibenzothiophene sulfone | 2.08 | 73 |

**1.3. Characterisation of hypercrosslinked polymers**

Fourier-transform infrared (FTIR) spectroscopy was carried out using a Bruker Tensor II spectrometer fitted with a Platinum ATR accessory (Bruker Optics). Samples were finely ground and analysed under ambient conditions. Spectra were collected over the range of 400-4000 cm^-1^ with a resolution of 4 cm^-1^, using 32 scans per measurement. Data processing employed a Blackman–Harris 3-term apodisation function and a zero-filling factor of 4. Throughout the measurements, the system was continuously purged with dry air. Spectral acquisition and analysis were performed using OPUS software (version 7.5).

Solid-state ^13^C NMR spectra were acquired on a Bruker Avance NEO 500 wide bore spectrometer (Bruker BioSpin), equipped with a 4 mm triple-resonance MAS probe. Approximately 15-25 mg of sample was packed into 4 mm zirconia rotors suitable for CRAMPS experiments. Spectra were recorded at a resonance frequency of 125.78 MHz, with the MAS spinning speed maintained at 14 kHz. Cross-polarisation was applied using a ramped contact pulse of 3 ms duration. Proton decoupling during acquisition was performed using the SPINAL sequence with 64 phase cycles. The 90° ^1^H pulse length was set to 2.5 µs, with a relaxation delay of 4 s. Around 2000 scans were averaged to achieve an adequate signal-to-noise ratio. ^13^C chemical shifts are given in ppm and were externally referenced to adamantane, assigning the downfield peak to 38.48 ppm.

X-ray photoelectron spectroscopy (XPS) analyses were conducted using a Nexsa Photoelectron Spectrometer (Thermo Scientific). High-resolution spectra were acquired at a pass energy of 50 eV and an energy step size of 0.1 eV. Measurements were carried out with monochromatic Al-Kα radiation (spot size: 400 µm). Data processing was performed using the Avantage software package (version 5.9931, Thermo Fisher Scientific). Elemental surface compositions were calculated based on peak areas, applying sensitivity factors from the ALTHERMO1 reference database.

Elemental analysis was performed using a Eurovector EA 3000 CHNS-O Elemental Analyser. Up to 3.0 mg of sample was accurately weighed into 4 × 6 mm tin capsules using a Sartorius ME 5 OCE microbalance and analysed in triplicate. Combustion and reduction temperatures were set to 1000 °C and 750 °C, respectively, while oxygen analysis was carried out at 1480 °C. High-purity helium (99.999%) served as the carrier gas throughout the measurements.

Nitrogen adsorption–desorption measurements were conducted at −196 °C (77 K) using a 3Flex surface characterisation analyser (Micromeritics). Prior to analysis, samples (50–100 mg) were degassed at 120 °C under vacuum for 14 hours using a VacPrep 061 unit (Micromeritics). Specific surface areas (SSAs) were calculated using the Brunauer–Emmett–Teller (BET) method, applied over the relative pressure range P/P₀ = 0.05–0.20. Pore size distributions (PSDs) were derived from the adsorption branch using the quenched solid density functional theory (QSDFT) model. Total pore volume (V_tot_) and micropore volume (V_mic_) were determined from the cumulative pore volumes calculated by the QSDFT model within the appropriate pore size ranges.

Water sorption isotherms were collected using a DVS-Resolution (Surface Measurement Systems). Around 10 mg of sample was weighed into a quartz crystal pan for measurement. Deionised water was used to generate the desired RH. Measurements began at 0% RH to remove residual water before being increased to the desired RH. Experiments were carried out at 25 °C. Isotherms were recorded up to 90% RH, using a step increment of 10% RH. Each step was equilibrated for 5 h, prior to measurement.

**1.4. Breakthrough measurements ((V)VOC capture)**

To estimate thermal stability and residual water content, each sorbent was thermogravimetrically analysed (Perkin Elmer, TGA 4000). The temperature program was initiated at 30 °C, held for 1 min, then heated to 800 °C at a rate of 20 °C min^-1^ under a constant N_2_ flow of 20 mL min^-1^.

To assess the degradation pattern of HCP-SO and HCP-S, around 20 mg of each analyte was separately introduced into a TGA (Perkin Elmer, TGA 4000) coupled to an FTIR gas cell (Thermo Scientific, Nicolet iZ10) with a transfer line heated to 250 °C and the cell heated to 300 °C. The TGA oven was first held at 30 °C for 1 min and then heated to 800 °C at a rate of 20 °C min^-1^. Emitted gaseous compounds were assessed via IR spectra recorded at a resolution of 4 cm^-1^ at wavenumbers of 4000 – 400 cm^-1^.

Glass liners (Gerstel, Twister Desorption Liner) were filled with dry sorbent, yielding a compact 4cm long sorbent layer in each case. Quartz wool (Roth, chemically clean) was used to hold the sorbent in place. To desorb potential contaminants from the synthesis, each glass liner was cleaned at 300 °C for 6 h under a constant N_2_ flow of ≥50 mL min^-1^.

Adsorption experiments (dry and humid) were performed once. A sum standard of 10 solvents (10 mol% each) was used comprising of ethanol, dichloromethane (DCM), acetone, ethyl acetate, 1-methoxypropan-2-yl acetate (MPA), *n*-decane, dihydrolevoglucosenone (Cyrene), acetonitrile, 2‑butoxyethyl acetate (EGBEA), and toluene. Solvents were chosen based on physicochemical properties that can be attributed to specific functionalities during adsorption behaviour, including Hansen solubility parameters, molar volume, log P, boiling point and vapour pressure. The apparatus for VOC capture varied depending on whether humid or dry conditions were used.

Under dry conditions, two sorbent columns and a headspace vial were connected in series (Figure S2). The headspace vial was cooled by an acetone-nitrogen_liq._ bath to condense analytes. A N_2_-flow was set at 50 mL min^-1^ and monitored with a bubble flow meter (Thermo Electron S.p.A., Soap film flow meter, 50 mL) before 0.5 µL of the sum standard was injected into the quartz wool. The flow was stopped after a purge volume of 5 L. The glass liners were detached and directly transferred to the thermodesorption-GC-MS. Headspace vials were exchanged after every 0.5 L of flow for fractionated analysis. Samples were then immediately measured by headspace-GC-MS.

Under humid conditions, two sorbent columns are connected in series (Figure S3). A dry and completely saturated air flow was mixed, resulting in 50% relative humidity (RH) as measured by a flow cell (Delta Ohm, HP480) and a datalogger (Delta Ohm, HD2101.5). A split flow of 50 mL min^-1^ was applied by an air pump (Gilian, Gil Air Plus) and directed toward the glass liners. After a total flow volume of 5 L, the pump was stopped, and the glass liners were detached and transferred directly to the thermodesorption-GC-MS.

All sorbent columns were automatically analysed by a Gerstel MultiPurpose Sampler, connected to a thermodesorption Unit (Gerstel, TDU). The thermodesorption oven was first heated from 30 °C to 300 °C at 720 °C min^-1^ and held for 5 min. Analytes were cryo-focused at -150 °C, then again heated to 300 °C at 720 °C min^-1^ and held for 5 min. The desorption flow was set to 50 mL min^-1^ for thermodesorption and 8 mL min^-1^ for cryo-focusing. All analytes were transferred to a GC (Thermo Finnigan, Trace GC) and separated using a Zebron ZB-624 column (30 m x 0.32 mm ID, film thickness 1.80 µm). The GC-oven was held at 35 °C for 10 min, then heated at 1 °C min^-1^ until 45 °C. The temperature was increased to 80 °C at 5 °C min^-1^ and finally to 250 °C at 20 °C min^-1^, where it was held for 10 min. Semi-quantitative analysis was performed using a mass spectrometer (Thermo Finnigan, Polaris Q) at a mass range of 15-500 m/z.

During headspace analysis, analytes were heated in an oven at 150 °C for 10 min in static mode, then transferred using an autosampler (Thermo Scientific, Triplus RSH) to a GC (Thermo Scientific, Trace GC Ultra)-MS (Thermo Scientific, ISQ). A Zebron ZB624 (30 m x 0.32 mm I.D., film thickness 1.80 µm) column was used. The GC-oven was first held at 40 °C for 1 min, then heated to 80 °C at 3 °C min^-1^. Lastly, the column was heated to 240 °C at 20 °C min^-1^. A split flow of 20 mL min^-1^ was used. Semi-quantitative analysis was performed at a mass range of 15-500 m/z.

Repeatability was tested by injecting an analyte mixture into the front column, which was coupled to a back column. Both columns were filled with commercial sorbent and the same ten analytes used in the standard are as described during dry adsorption experiments. Additionally, the same analytical procedure described for adsorption experiments was employed. The procedure was repeated three times.

To confirm the regenerability of HCP-N, 0.5 µL of the described standard mixture was injected into an activated front column under a constant nitrogen flow of 50 mL min^-1^. After a purge volume of 5 L, the column was immediately analysed by TD (Gerstel, TDU 2) -GC (Agilent, 8860 GC System), -MS (Agilent, 5977C GC/MSD). To separate the analytes, a HP – 5MS UI column from Agilent J&W (30 m × 0.25 mm I.D., film thickness 0.25 µm) was used. The split flow was set to 100 mL min^-1^ during analyte desorption and after cryo-focusing. The same TD-GC-MS method was applied as described for sampling under dry conditions. After each run the column was activated at 300°C for 1 h. The procedure was repeated five times.

**
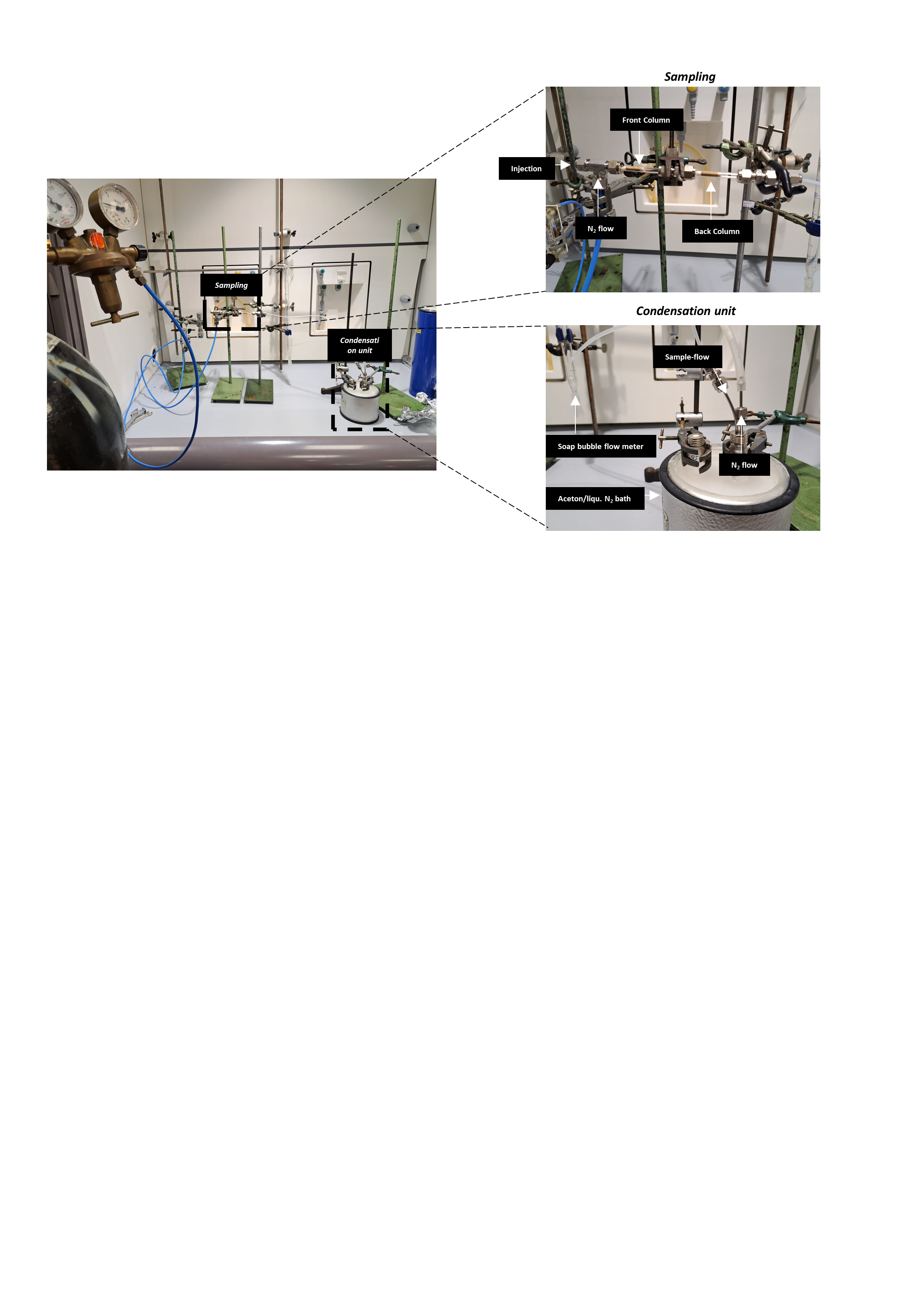
**

**Figure S2.** Apparatus for (V)VOC analysis under dry inert conditions.


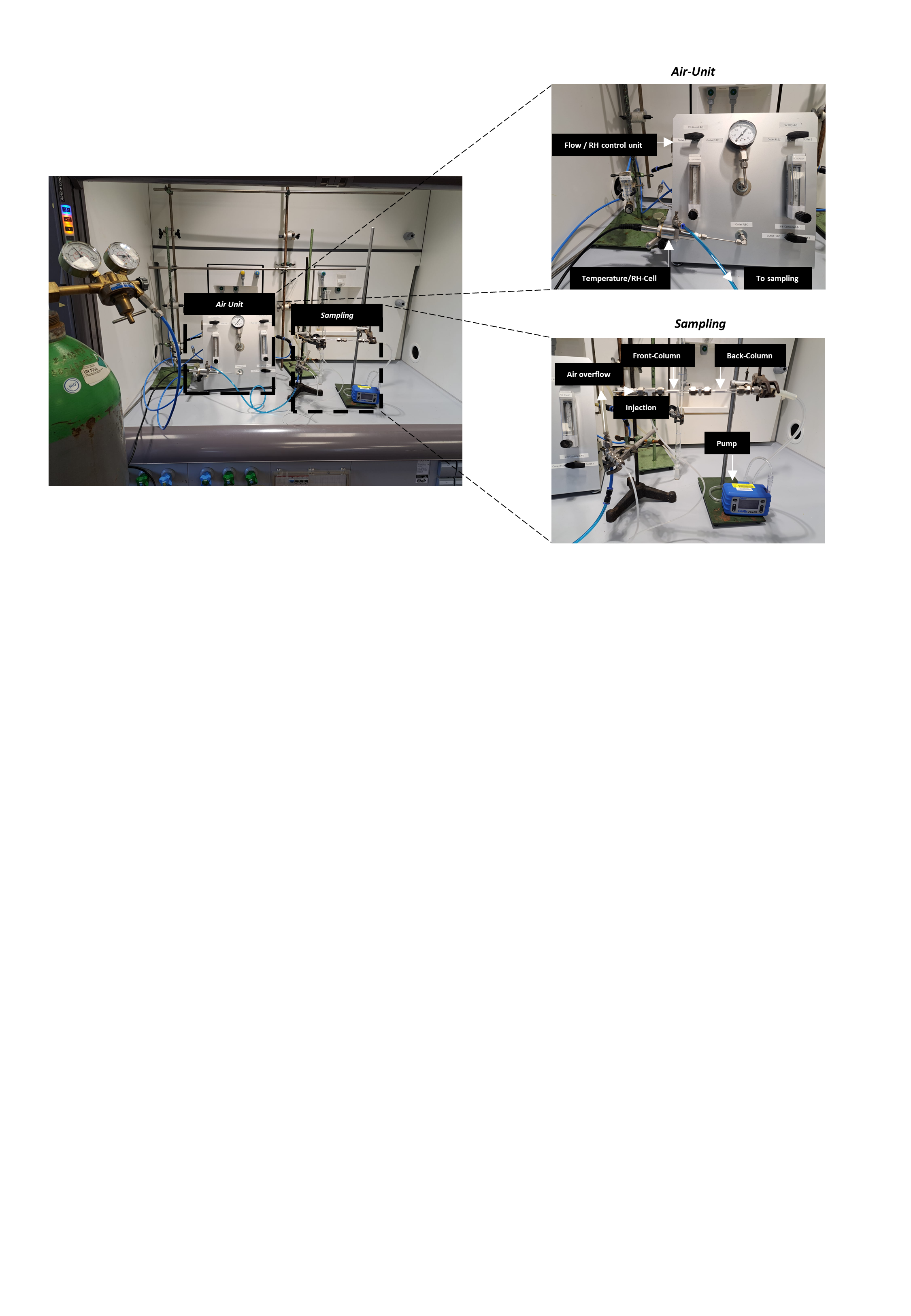


**Figure S3.** Apparatus for (V)VOC measurements under humid conditions.

**2. Supporting figures and tables**


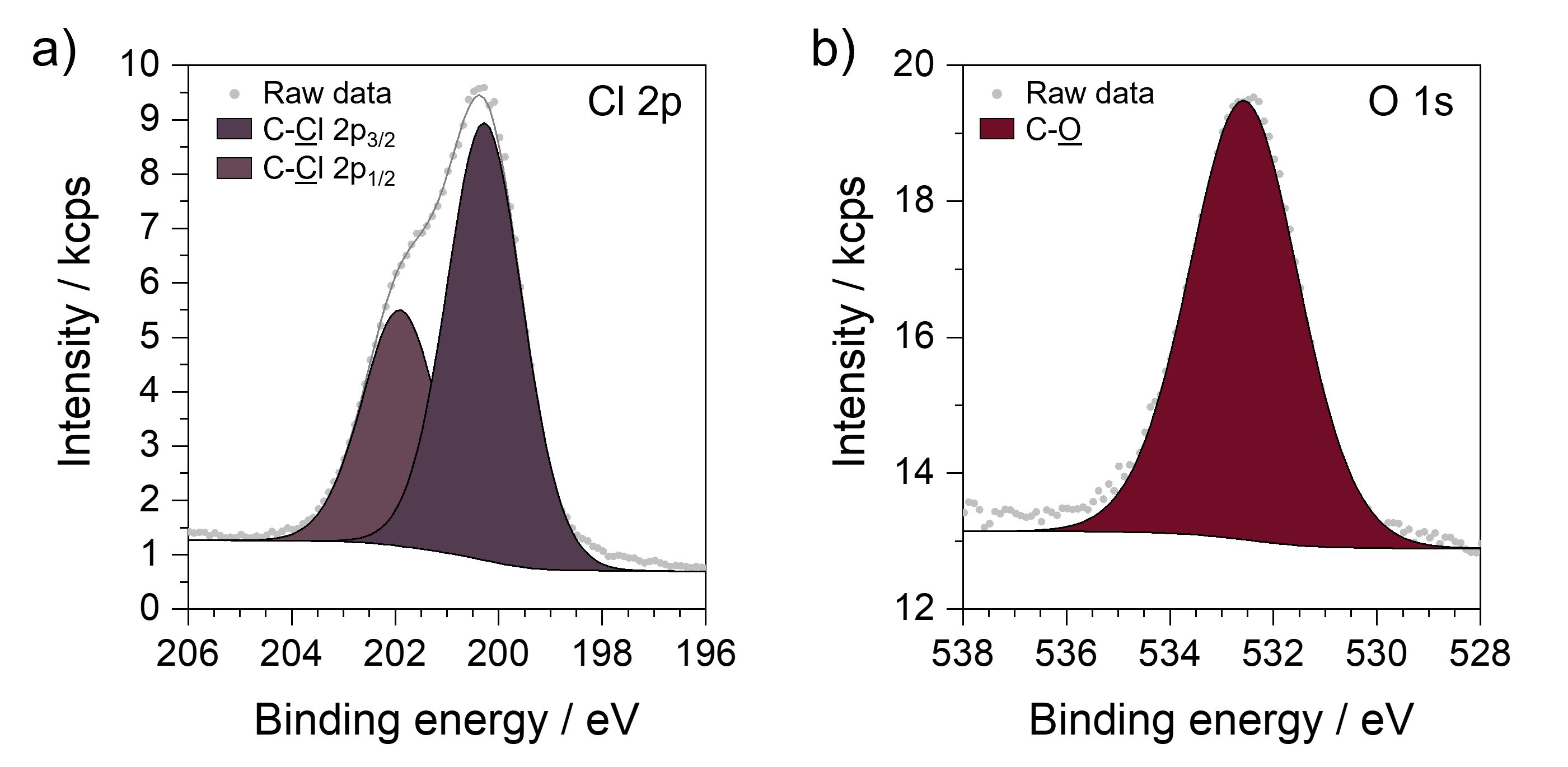


**Figure S4.** X-ray photoelectron spectroscopy spectra of HCP-C. a) High-resolution Cl 2p spectrum, and b) High-resolution O 1s spectrum.


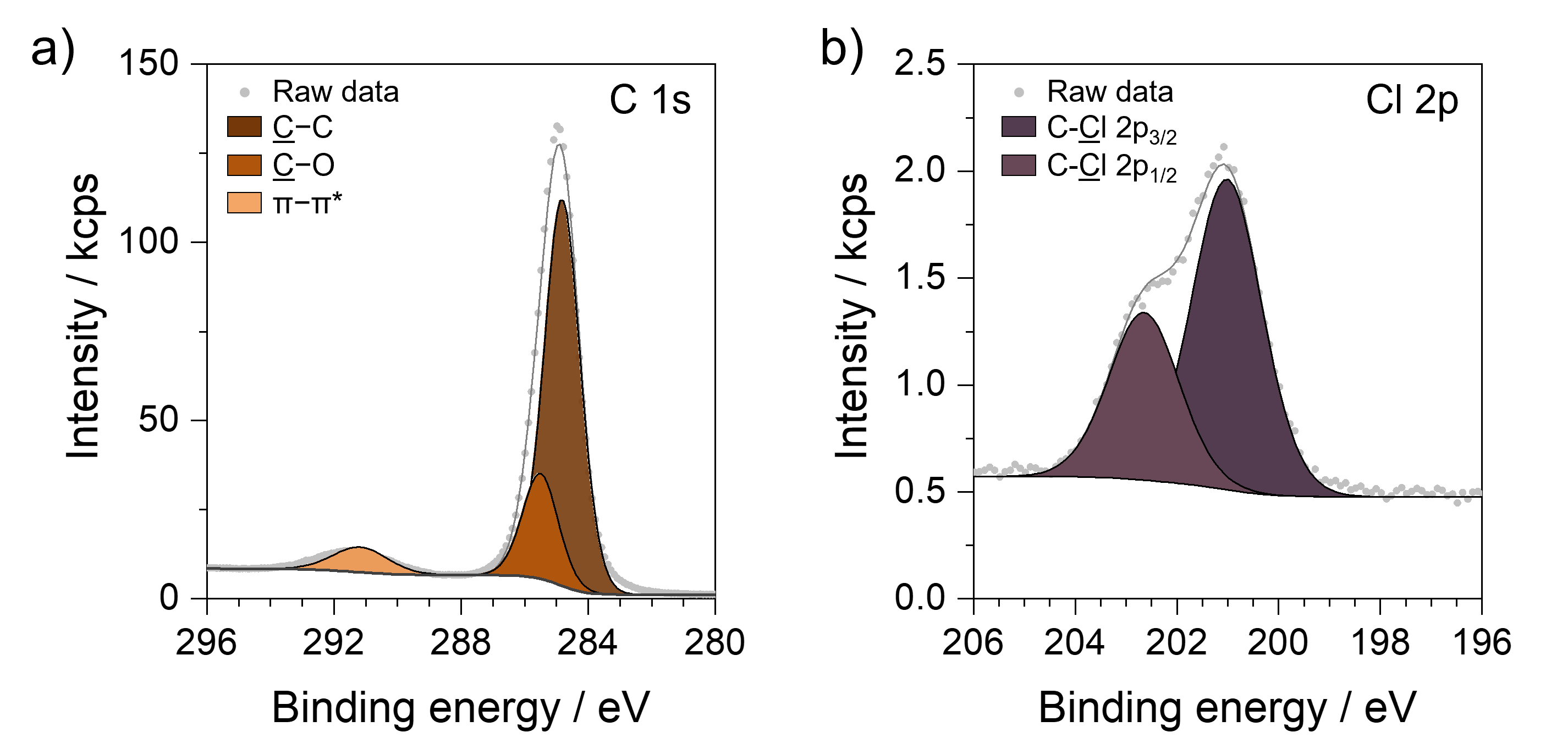


**Figure S5.** X-ray photoelectron spectroscopy spectra of HCP-N. a) High-resolution C 1s spectrum, and b) High-resolution Cl 2p spectrum.


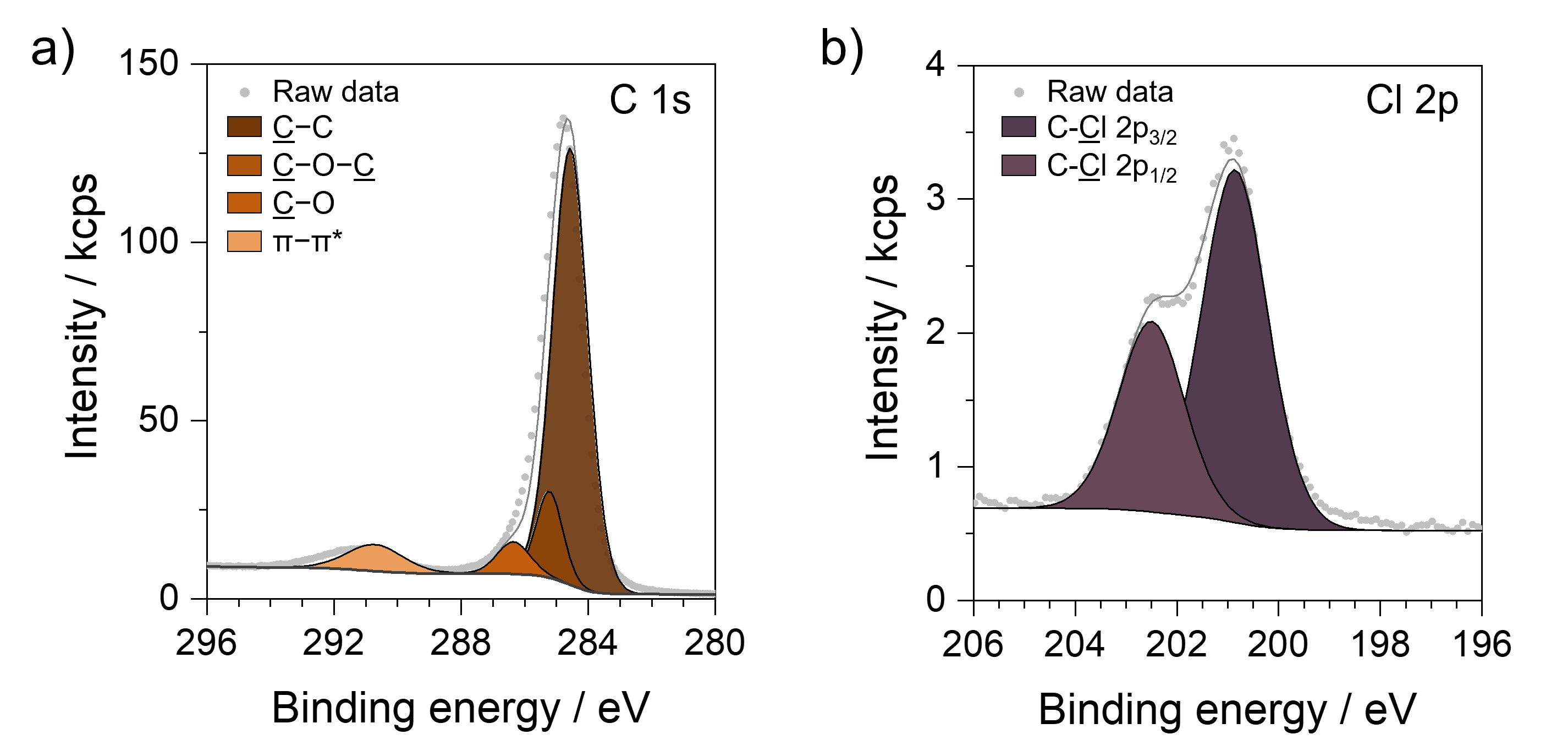


**Figure S6.** X-ray photoelectron spectroscopy spectra of HCP-O. a) High-resolution C 1s spectrum, and b) High-resolution Cl 2p spectrum.


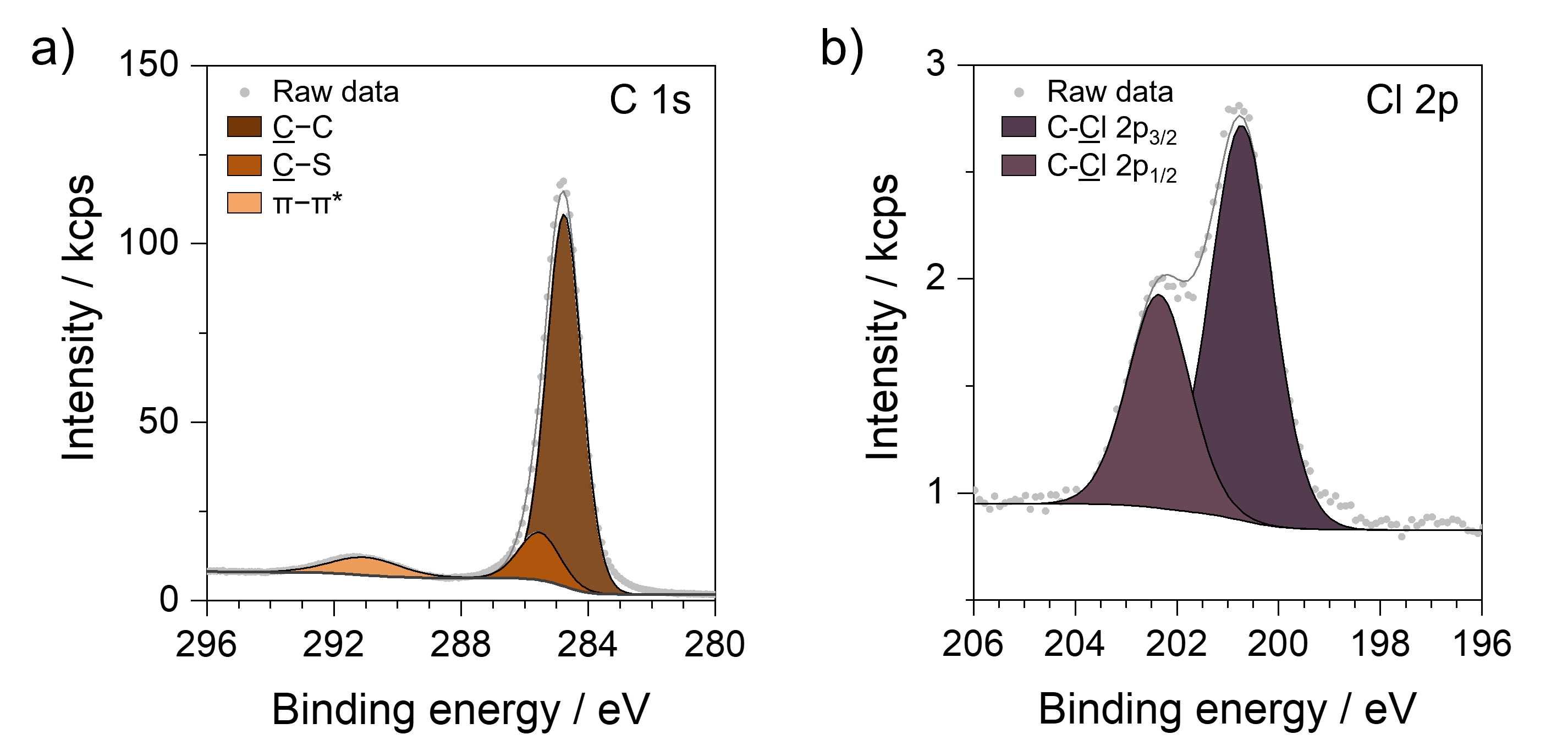


**Figure S7.** X-ray photoelectron spectroscopy spectra of HCP-S. a) High-resolution C 1s spectrum, and b) High-resolution Cl 2p spectrum.


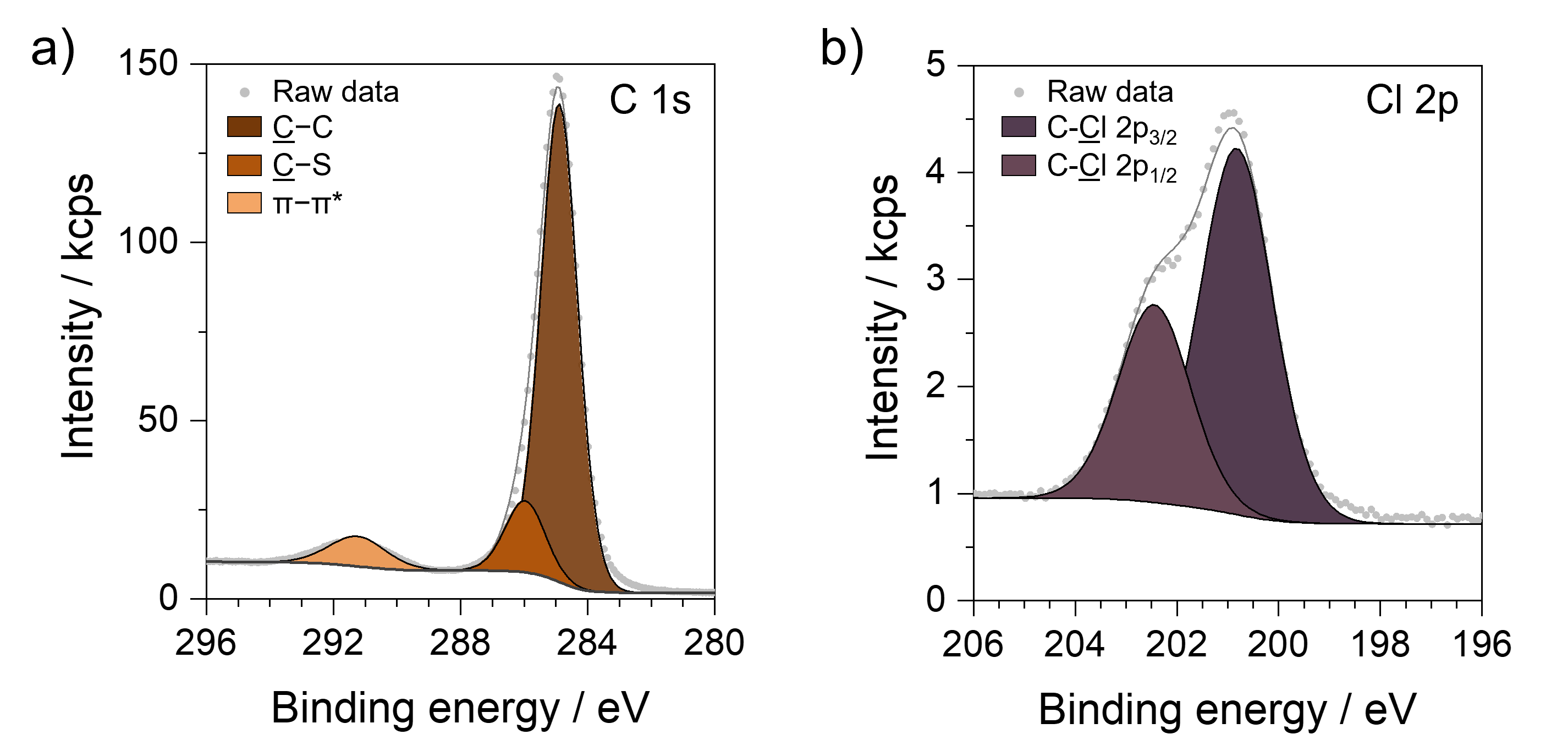


**Figure S8.** X-ray photoelectron spectroscopy spectra of HCP-SO. a) High-resolution C 1s spectrum, and b) High-resolution Cl 2p spectrum.

**Table S2**. Elemental composition of biphenyl-based HCPs determined by XPS.

|  | C (wt.%) | N (wt.%) | O (wt.%) | S (wt.%) | Cl (wt.%) |
| --- | --- | --- | --- | --- | --- |
| HCP-C | 87.44 ± 0.25 | - | 3.71 ± 0.13 | - | 8.87 ± 0.37 |
| HCP-N | 95.06 ± 0.06 | 1.80 ± 0.03 | 1.41 ± 0.02 | - | 1.75 ± 0.06 |
| HCP-O | 93.63 ± 0.12 | - | 3.54 ± 0.12 | - | 2.84 ± 0.02 |
| HCP-S | 92.57 ± 0.08 | - | 1.82 ± 0.24 | 3.39 ± 0.07 | 2.23 ± 0.09 |
| HCP-SO | 91.91 ± 0.03 | - | 3.32 ± 0.12 | 3.52 ± 0.11 | 1.25 ± 0.01 |

**Table S3**. Elemental composition of non-sulfonated HCPs determined by EA.

|  | C (wt.%) | N (wt.%) | O (wt.%) | S (wt.%) | H (wt.%) |
| --- | --- | --- | --- | --- | --- |
| HCP-C | 83.74 ± 0.21 | - | 3.16 ± 0.31 | - | 4.85 ± 0.02 |
| HCP-N | 88.91 ± 0.49 | 2.89 ± 0.08 | 1.01 ± 0.23 | - | 5.22 ± 0.05 |
| HCP-O | 89.48 ± 0.91 | - | 3.70 ± 0.05 | - | 5.02 ± 0.08 |
| HCP-S | 85.37 ± 1.75 | - | 0.97 ± 0.11 | 5.49 ± 0.20 | 4.70 ± 0.21 |
| HCP-SO | 88.90 ± 1.36 | - | 1.93 ± 0.12 | 3.76 ± 0.12 | 5.18 ± 0.08 |

**
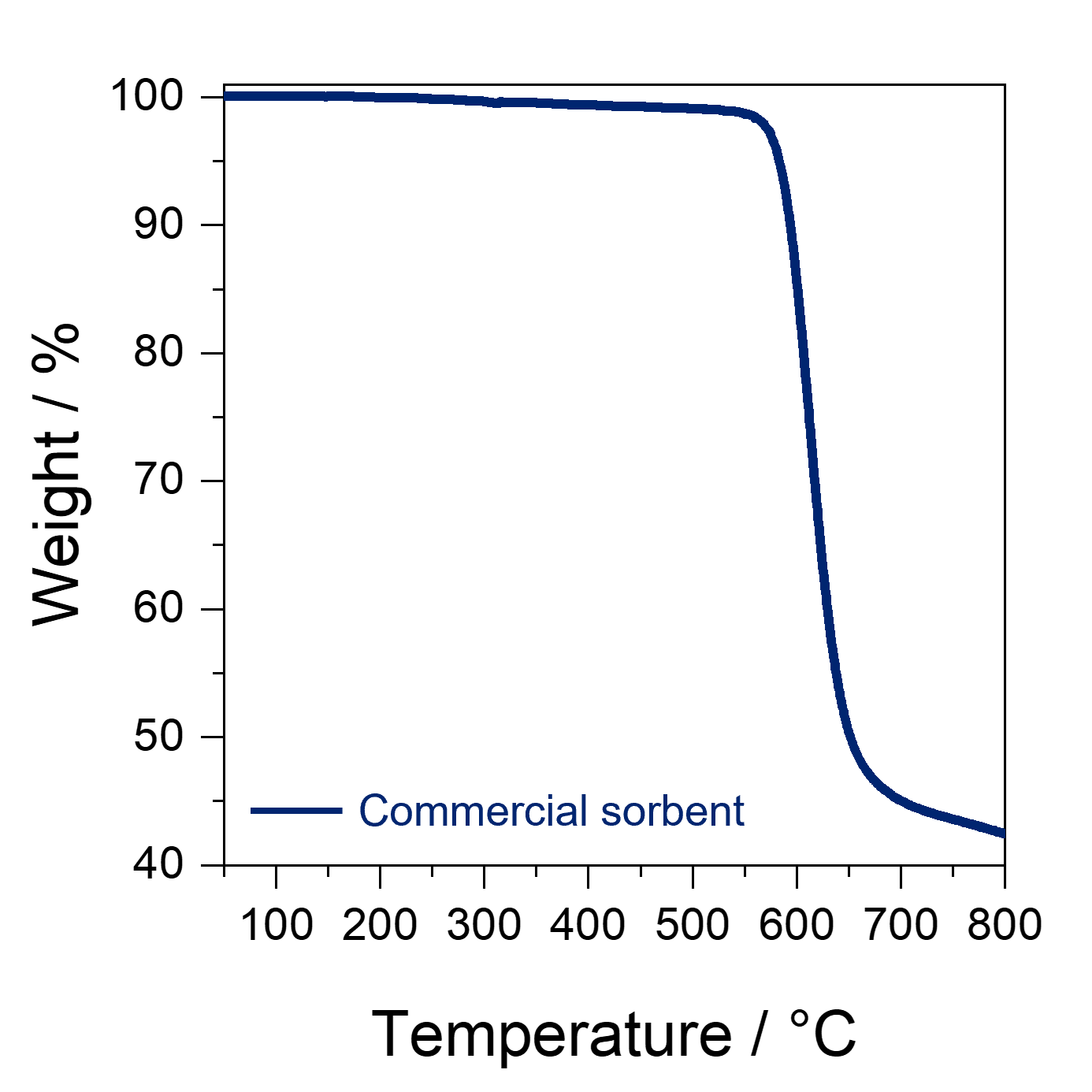
**

**Figure S9.** TGA data of the commercial sorbent.

**Table S4.** Physicochemical properties of analytes (DCM: dichloromethane, EGBEA: 2-butoxy ethylacetate and MPA: methoxy propylacetate) and sorbents for comparison with VVOC measurements.

| Analyte / Sorbent | δ_D_  (MPa^0.5^) | δ_P_  (MPa^0.5^) | δ_H_  (MPa^0.5^) | Log P | Molar volume  (mL mol^-1^) | Boiling point  (°C) | Vapor Pressure  (25 °C) (mPa) | Molar weight  (g mol^-1^) |
| --- | --- | --- | --- | --- | --- | --- | --- | --- |
| Acetone | 15.5 | 10.4 | 7 | -0.24 | 738 | 56 | 232 | 58 |
| Acetonitrile | 15.3 | 18 | 6.1 | -0.34 | 52.9 | 82 | 86.6 | 41 |
| Cyrene | 18.9 | 12.4 | 7.1 | -0.20 | 102.5 | 226 | 0.094 | 128 |
| Decane | 15.5 | 0.1 | 0 | 5.01 | 160 | 174 | 1.26 | 142 |
| EGBEA | 15.3 | 7.5 | 6.8 | 1.51 | 171 | 192 | 0.228 | 160 |
| Ethanol | 15.8 | 8.8 | 19.4 | -0.31 | 58.6 | 78 | 59.1 | 46 |
| Ethyl acetate | 15.8 | 5.3 | 7.2 | 0.73 | 98.6 | 77 | 96.5 | 88 |
| DCM | 17 | 7.3 | 7.1 | 1.25 | 64.4 | 40 | 436 | 84 |
| MPA | 15.6 | 5.6 | 9.8 | 0.56 | 137 | 146 | 3.15 | 132 |
| Toluene | 18 | 2.4 | 2 | 2.73 | 106.6 | 111 | 28.4 | 92 |
| HCP-C | 20 | 2.8 | 2.8 | - | - | - | - | - |
| HCP-O | 19.7 | 4 | 3.2 | - | - | - | - | - |
| HCP-S | 20.4 | 3.3 | 4.5 | - | - | - | - | - |
| HCP-N | 20.8 | 6 | 5.4 | - | - | - | - | - |
| HCP-SO | 20.8 | 12.9 | 5.6 | - | - | - | - | - |


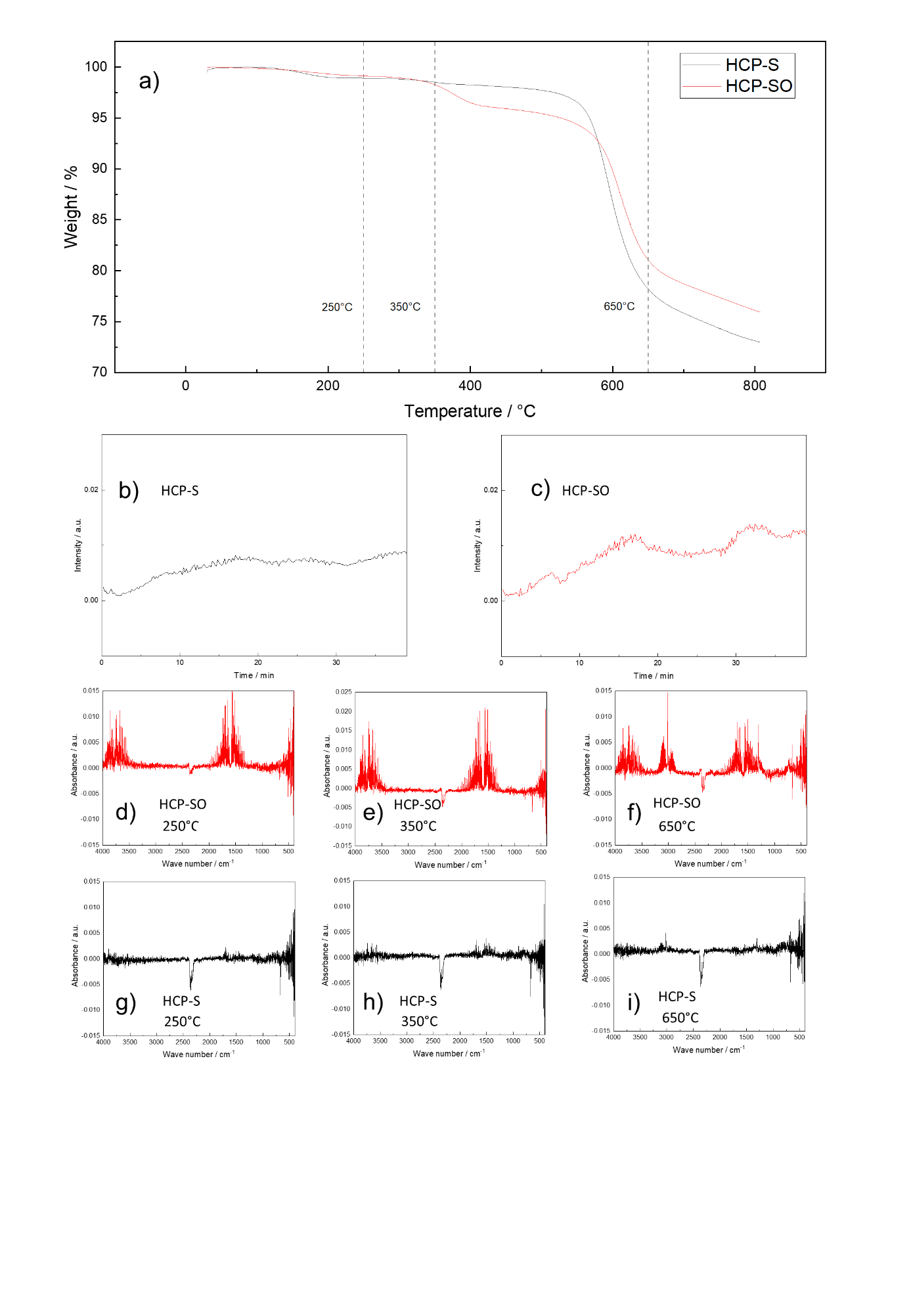


**Figure S10.** TGA-FTIR data of HCP-SO and HCP-S. TGA thermograms of HCP-SO and -S are depicted in graph a). Cumulative FTIR signals were recorded from 30 °C to 800 °C and are shown for HCP-S in graph b) and for HCP-SO in graph c). In graph d-i), FTIR spectra at specific weight loss steps (250°C, 350°C and 650°C) are presented for HCP-SO and HCP-S.


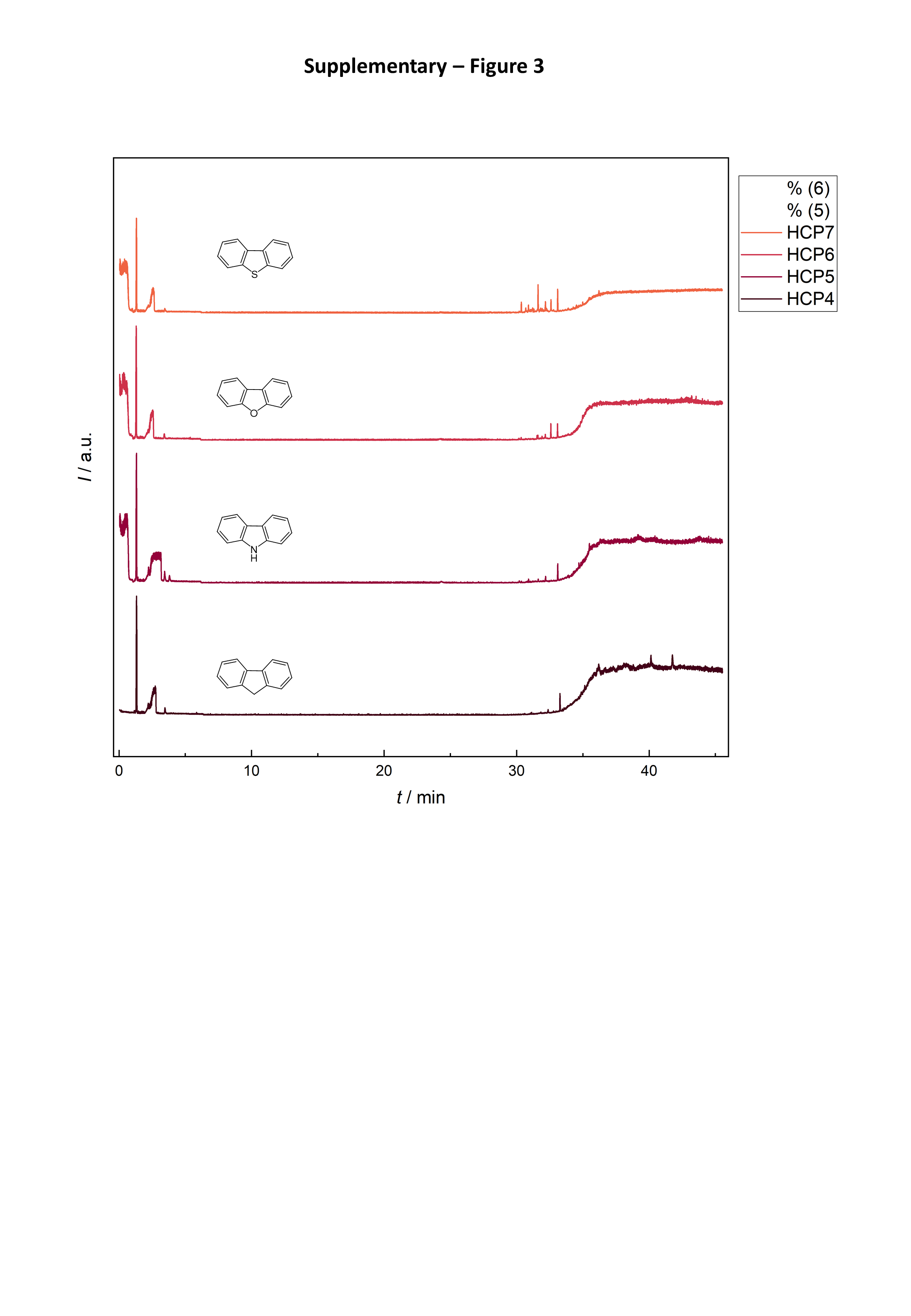


**Figure S11.** Thermodesorption-GC-MS measurements directly after the sorbent cleaning in TIC mode.


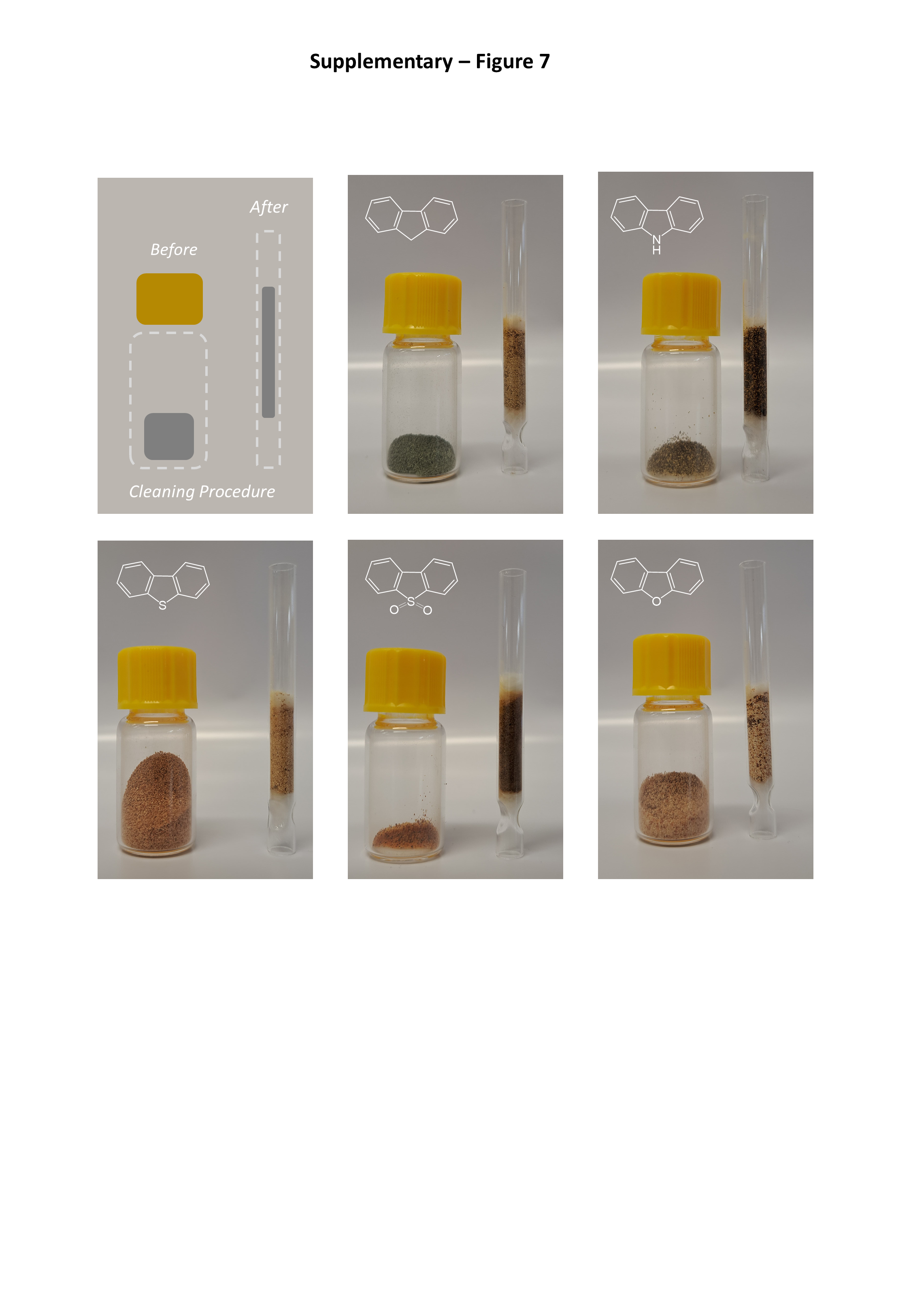


**Figure S12.** Pictures of hypercrosslinked polymers before (glass bottle with yellow cap) and after (glass liner) cleaning procedure.


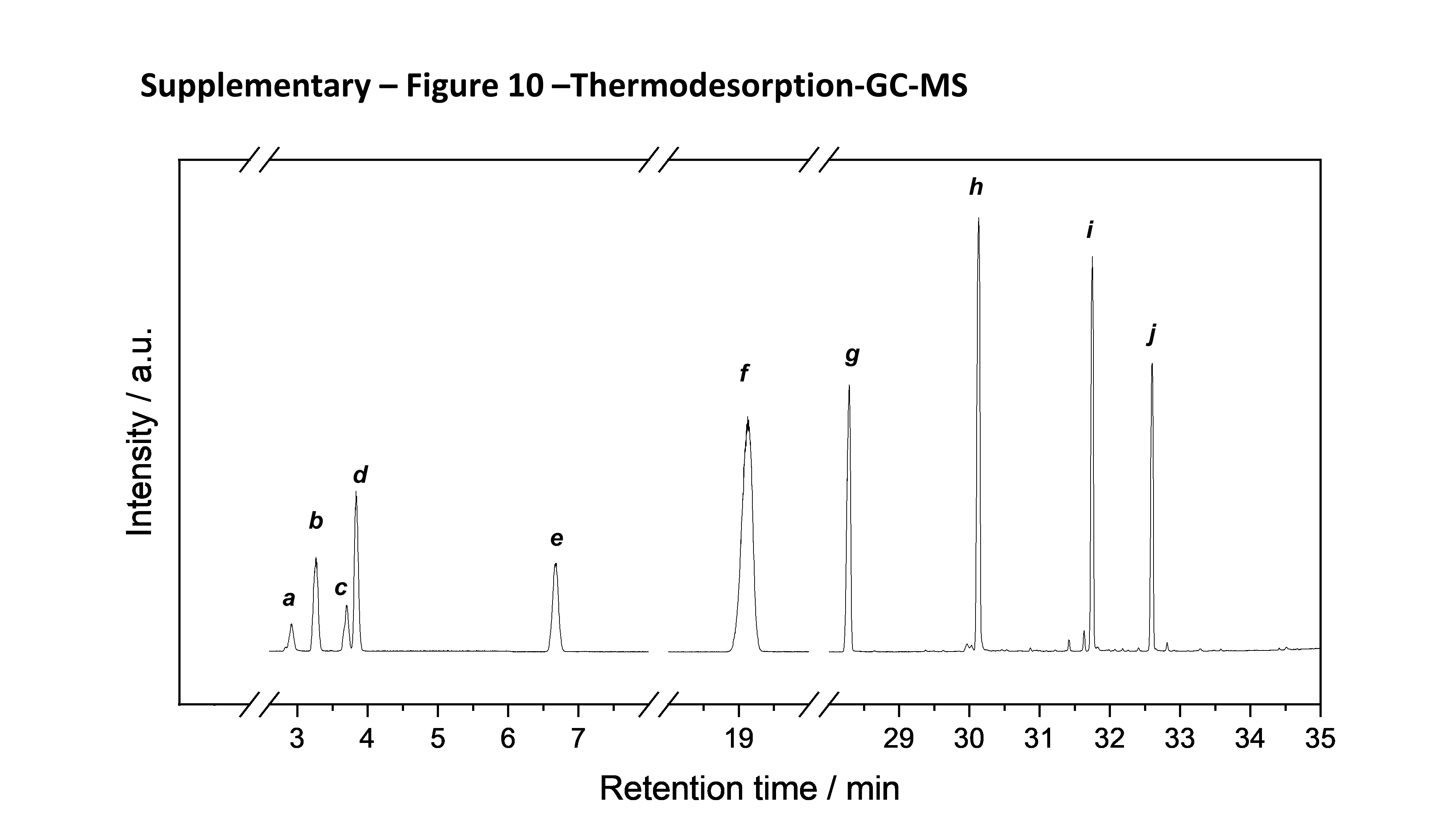


**Figure S13.** Exemplary thermodesorption-GC-MS chromatogram (*a*: ethanol, *b*: acetone, *c*: acetonitrile, *d*: dichloromethane, e: ethyl acetate, f: toluene, g: 1-methoxypropan-2-yl acetate, h: decane, i: 2-butoxyethyl acetate, *j*: dihydrolevoglucosenone) of the breakthrough analysis of HCP-N under inert conditions.


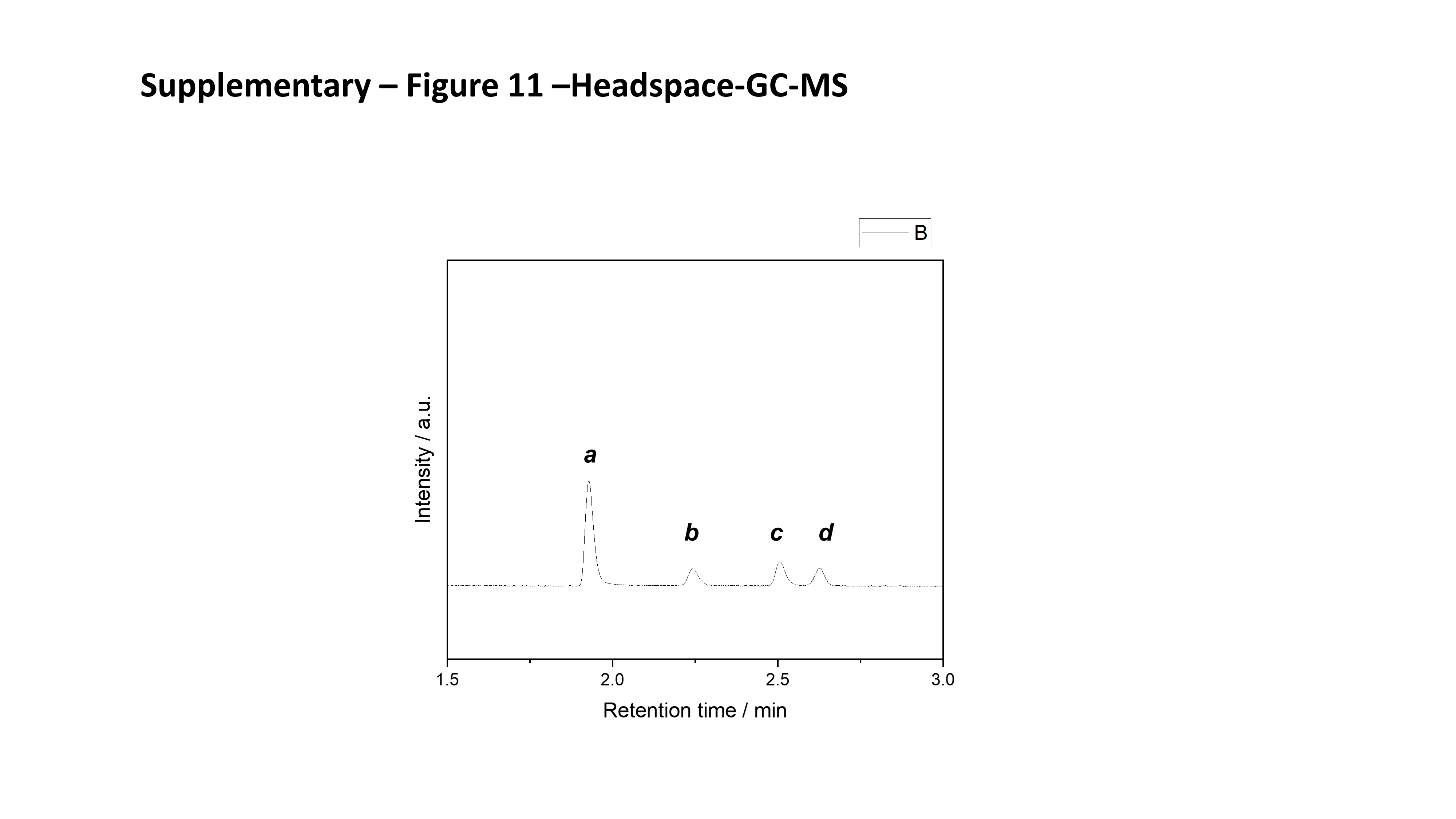


**Figure S14.** Exemplary headspace-GC-MS data of the commercial sorbent at 0.5 L breakthrough volume (*a*: ethanol, *b*: acetone, *c*: acetonitrile, *d*: dichloromethane).


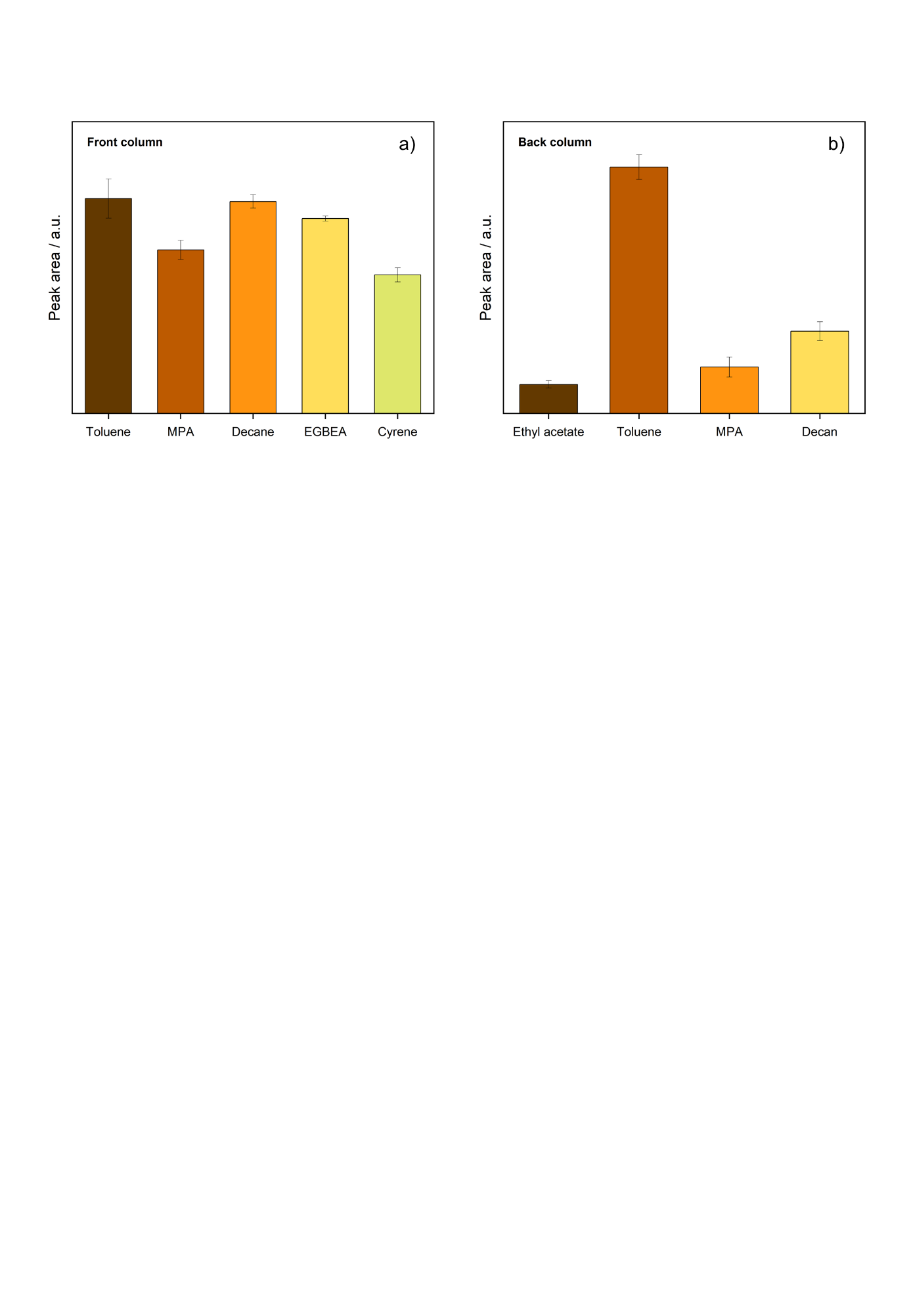


**Figure S15.** Repeatability tests (*n*=3) were performed with the commercial sorbent. Analytes were injected into the front column a) coupled to a back column b) and analysed with TD-GC-MS. The analytes ethanol, dichloromethane, acetonitrile, and acetone were not retained by the commercial sorbent. Abbreviations used: MPA: 1-methoxy-2-propylacetate, EGBEA: butylglycolacetate.


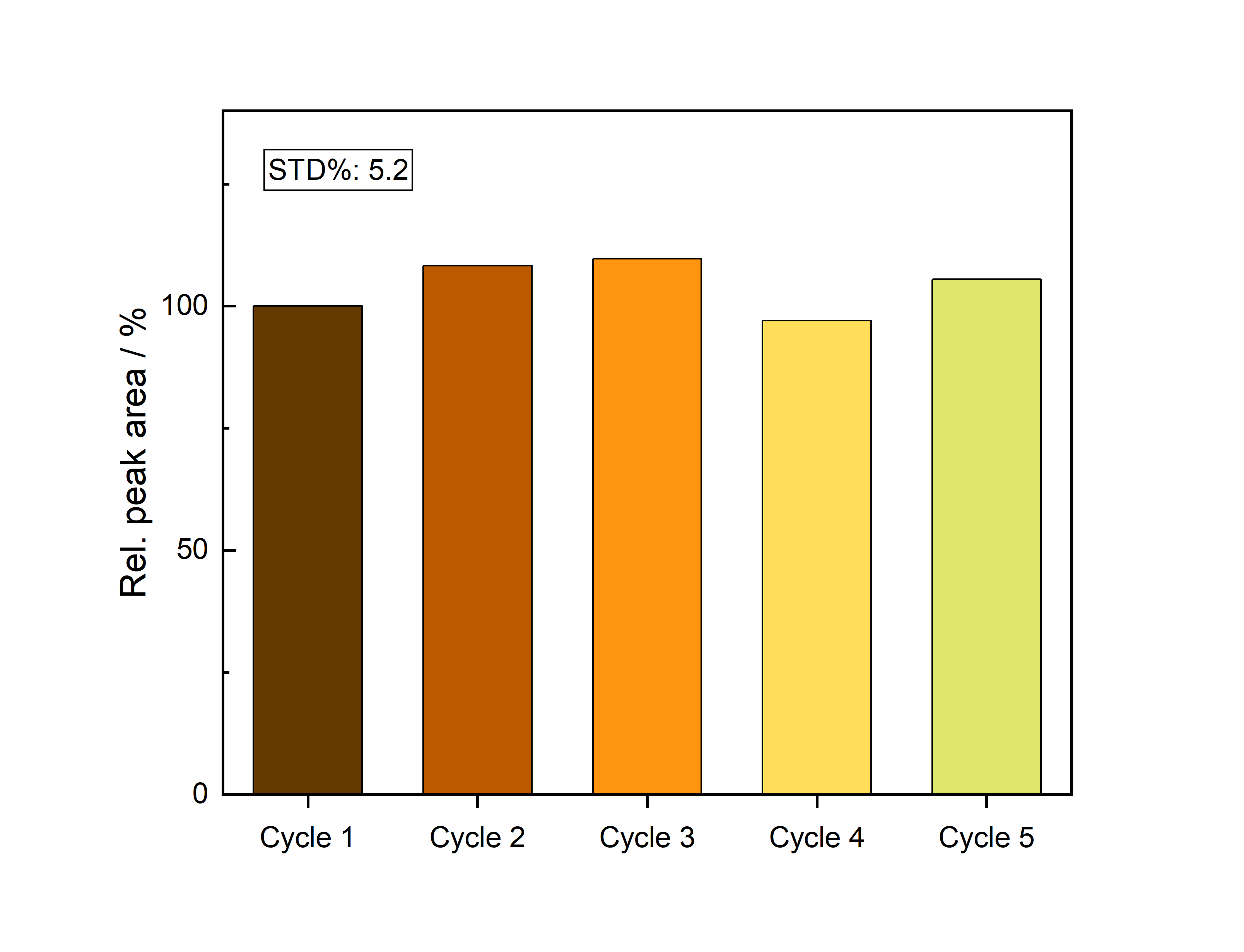


**Figure S16.** Regenerability experiments assessed by TD-GC-MS of HCP-N demonstrated with the analyte cyrene, yielding low standard deviations (5.2%) across five cycles.


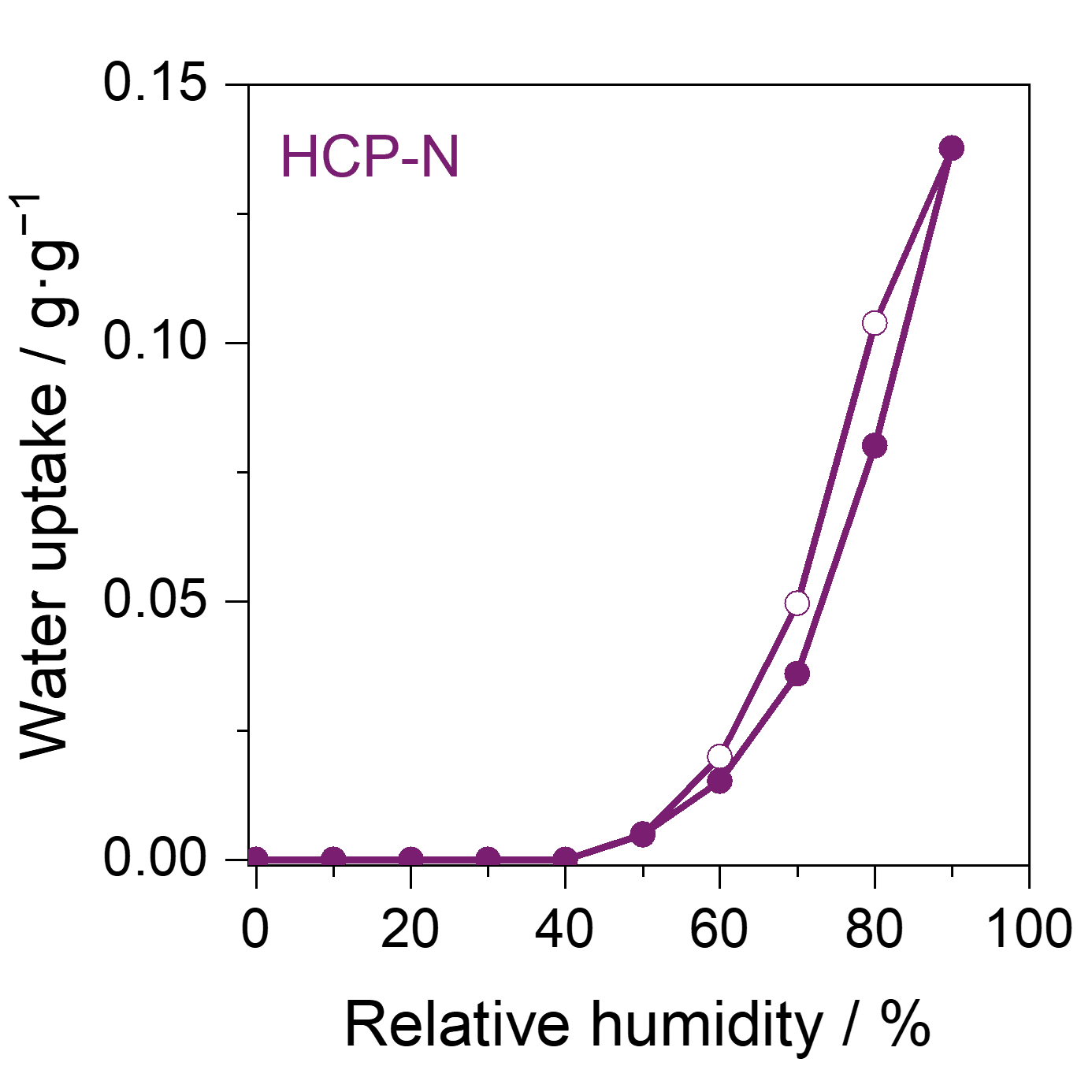


**Figure S17.** Water isotherm of HCP-N at 25 °C. Closed symbols indicate adsorption and open symbols, desorption.

**Table S5.** Comparison of adsorbent performance for capture of mixed (V)VOC streams. Dashes represent data not provided in the literature. The adsorption of 1-methoxypropan-2-yl acetate (MPA), 2-butoxyethyl acetate, and cyrene was excluded from the comparison as they were not found in the literature. Analytical procedure used for the determination of breakthrough: ^a^TD-GC-MS; ^b^Solvent extraction / GC-MS. This table is only for comparative purposes, since the experimental procedure for the determination of breakthrough can vary with the respective reference.

| Sorbent | Breakthrough of analyte (%) | | | | | | | Ref. in Publication |
| --- | --- | --- | --- | --- | --- | --- | --- | --- |
|  | Ethanol | Acetone | Acetonitrile | DCM | Ethyl acetate | Toluene | Decane |  |
| HCP-C | 78.3 | 8.9 | 84.7 | 51.8 | 0.0 | 0.0 | 0.0 | This work^a^ |
| HCP-N | 0.0 | 9.1 | 0.0 | 0.0 | 0.0 | 0.8 | 0 | This work^a^ |
| HCP-N (50% RH) | 0.0 | 0.0 | 0.0 | 0.0 | 0.0 | 0.0 | 0.0 | This work^a^ |
| HCP-O | 100 | 23.5 | 71.2 | 100 | 0.0 | 0.5 | 0.0 | This work^a^ |
| HCP-S | 100 | 0.0 | 100 | 100 | 0.0 | 0.0 | 0.0 | This work^a^ |
| Tenax TA | 83 ± 6 | 62 ± 2 | 75 ± 12 | - | 12 ± 3 | 0.0 (Benzene) | 15 ± 5 (Hexane) | (23)^a^ |
| Tenax GR | 27 ± 5 | 0.0 | 29 ± 4 | - | 13 ± 1 | 0.0 | 3 ± 0 | (23)^a^ |
| Carbograph 5TD | 34 ± 13 | 0.0 | 25 ± 3 | - | 24 ± 10 | 0.0 | 2 ± 1 | (23)^a^ |
| Carbopack B | 32 ± 4 | 0.0 | 38 ± 15 | - | 32 ± 15 | 0.0 | 2 ± 1 | (23)^a^ |
| Carbopack Z | 59 ± 3 | 9 ± 18 | 33 ± 5 | - | 72 ± 12 | 0.0 | 2 ± 1 | (23)^a^ |
| Carbosieve SII | 17 ± 4 | 1 ± 3 | 20 ± 12 | - | 19 ± 7 | 0.0 | 7 ± 5 | (23)^a^ |
| Carboxen 569 | 20 ± 6 | 0.0 | 24 ± 6 | - | 14 ± 1 | 0.0 | 2 ± 1 | (23)^a^ |
| Carboxen 1003 | 21 ± 2 | 4 ± 4 | 32 ± 13 | - | 16 ± 4 | 0.0 | 4 ± 4 | (23)^a^ |
| Carboxen 1018 | 23 ± 7 | 2 ± 3 | 26 ± 1 | - | 16 ± 3 | 0.0 | 6 ± 3 | (23)^a^ |
| Carbotrap 300 | 23 ± 2 | 0.0 | 1 ± 2 | - | 8 ± 1 | 3 ± 1 | 0.0 | (23)^a^ |
| Sibata carbon beads | 31 ± 2 (1-Butanol) | 12 ± 6 | - | 8 ± 13 | 2 ± 8 | 0.0 | 0.0 (Hexane) | (32)^b^ |
| Anasorb CSC | 38 ± 11 | 16 ± 10 | - | 0.0 | 9 ± 8 | 0.0 | 3 ± 5 | (32)^b^ |
| Bead-shaped activated carbon | 2 ± 7 | 16 ± 14 | - | 0.0 | 0.0 | 0.0 | 0.0 | (32)^b^ |
| ZIF-7 | - | - | - | - | 56.7 (Butyl acetate) | 55.8 | 46.1 (Undecane) | (37)^a^ |
| Activated carbon |  |  |  |  | 63.0 | 50.3 | 55.9 | (37)^a^ |
| Tenax TA | 40.1 ± 38.5 | - | - | - | - | - | - | (38)^a^ |
| Carbograph 5TD | 29.1 ± 24.5 | - | - | - | - | - | - | (38)^a^ |
| Carbopack X | 69.7 ± 31.2 | - | - | - | - | - | - | (38)^a^ |
| Carbotrap X | 6.3 ± 0.8 | - | - | - | - | - | - | (38)^a^ |

**Table S6.** Estimated cost price per gram of HCPs and a variety of commercial sorbents for (V)VOC capture. A description of the estimated cost calculations is outlined below, and the cost of commercial sorbents was sourced from suppliers.

| (V)VOC adsorbent | Estimated cost (€·g^–1^) | Source |
| --- | --- | --- |
| HCP-C | 1.12 | This work |
| HCP-N | 1.29 | This work |
| HCP-O | 1.10 | This work |
| HCP-S | 1.66 | This work |
| HCP-SO | 2.90 | This work |
| TENAX TA | 35.90 | Commercial * |
| TENAX GR | 32.00 | Commercial * |
| Carbopack B | 63.60 | Commercial * |
| Carbopack X | 63.60 | Commercial * |
| Carbosieve S-II | 63.60 | Commercial * |
| Carboxen 569 | 4.66 | Commercial * |
| Carbotrap X | 67.60 | Commercial * |
| Graphitised carbon black | 32.80 | Commercial * |
| Carboxen 1003 | 62.60 | Commercial * |
| ZSM-5 (Zeolite) | 18.37 | Commercial * |
| * Price sourced from Sigma-Aldrich (subsidiary of Merck KGaA) as of August 1^st^, 2025 | | |

**Cost estimation for Table S6:** Estimated cost per gram of HCPs for (V)VOC capture are calculated and compared to commercial competitors, the performance of some of which is provided in Table S5. Estimated costs were calculated using the reagents required for their synthesis. The lowest unit price for 100 g (solids) or 1 L (liquids) found on Merck (www.sigmaaldrich.com/AT/de) as of 1^st^ of August 2025 was used and adjusted to adhere to the amount required for synthesis. A minimum reagent purity of ≥98% was used. If reagents fulfilling these criteria were not available, the next closest option to the aforementioned requirements was used. Work-up steps, such as washing, were excluded from the calculations. Labour, synthesis time, and power were also not factored into the calculation. A detailed breakdown of the estimated costs is provided in an accompanying file. The cost of 1 g of commercial materials available from the same supplier (Merck) was derived from the lowest unit price for 100 g, if available. We would like to reiterate that the estimated cost provided of HCPs is exclusively for comparative purposes and only considers lab-scale production.
